# Structure-Guided Design of a Domain-Selective Bromodomain and Extra Terminal N-Terminal Bromodomain Chemical Probe

**DOI:** 10.1021/acs.jmedchem.3c00906

**Published:** 2023-11-15

**Authors:** Erin Bradley, Lucia Fusani, Chun-wa Chung, Peter D. Craggs, Emmanuel H. Demont, Philip G. Humphreys, Darren J. Mitchell, Alex Phillipou, Inmaculada Rioja, Rishi R. Shah, Christopher R. Wellaway, Rab K. Prinjha, David S. Palmer, William J. Kerr, Marc Reid, Ian D. Wall, Rosa Cookson

**Affiliations:** † GSK, Medicines Research Centre, Stevenage SG1 2NY, Hertfordshire, U.K.; ‡ Department of Pure and Applied Chemistry, University of Strathclyde, Thomas Graham Building, 295 Cathedral Street, Glasgow G1 1XL, U.K.

## Abstract

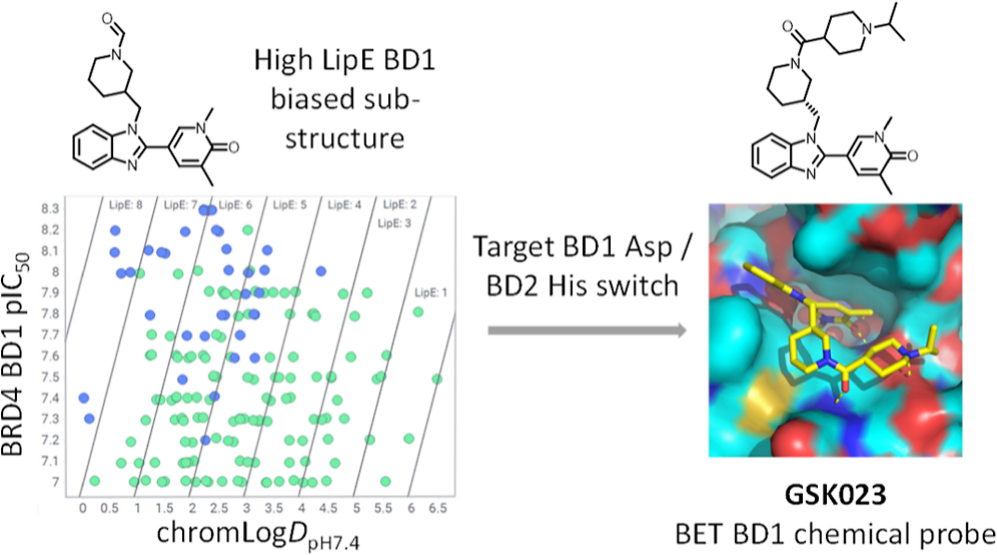

Small-molecule-mediated disruption of the protein–protein interactions between acetylated histone tails and the tandem bromodomains of the bromodomain and extra-terminal (BET) family of proteins is an important mechanism of action for the potential modulation of immuno-inflammatory and oncology disease. High-quality chemical probes have proven invaluable in elucidating profound BET bromodomain biology, with seminal publications of both pan- and domain-selective BET family bromodomain inhibitors enabling academic and industrial research. To enrich the toolbox of structurally differentiated N-terminal bromodomain (BD1) BET family chemical probes, this work describes an analysis of the GSK BRD4 bromodomain data set through a lipophilic efficiency lens, which enabled identification of a BD1 domain-biased benzimidazole series. Structure-guided growth targeting a key Asp/His BD1/BD2 switch enabled delivery of GSK023, a high-quality chemical probe with 300–1000-fold BET BD1 domain selectivity and a phenotypic cellular fingerprint consistent with BET bromodomain inhibition.

## Introduction

**Figure 1:**
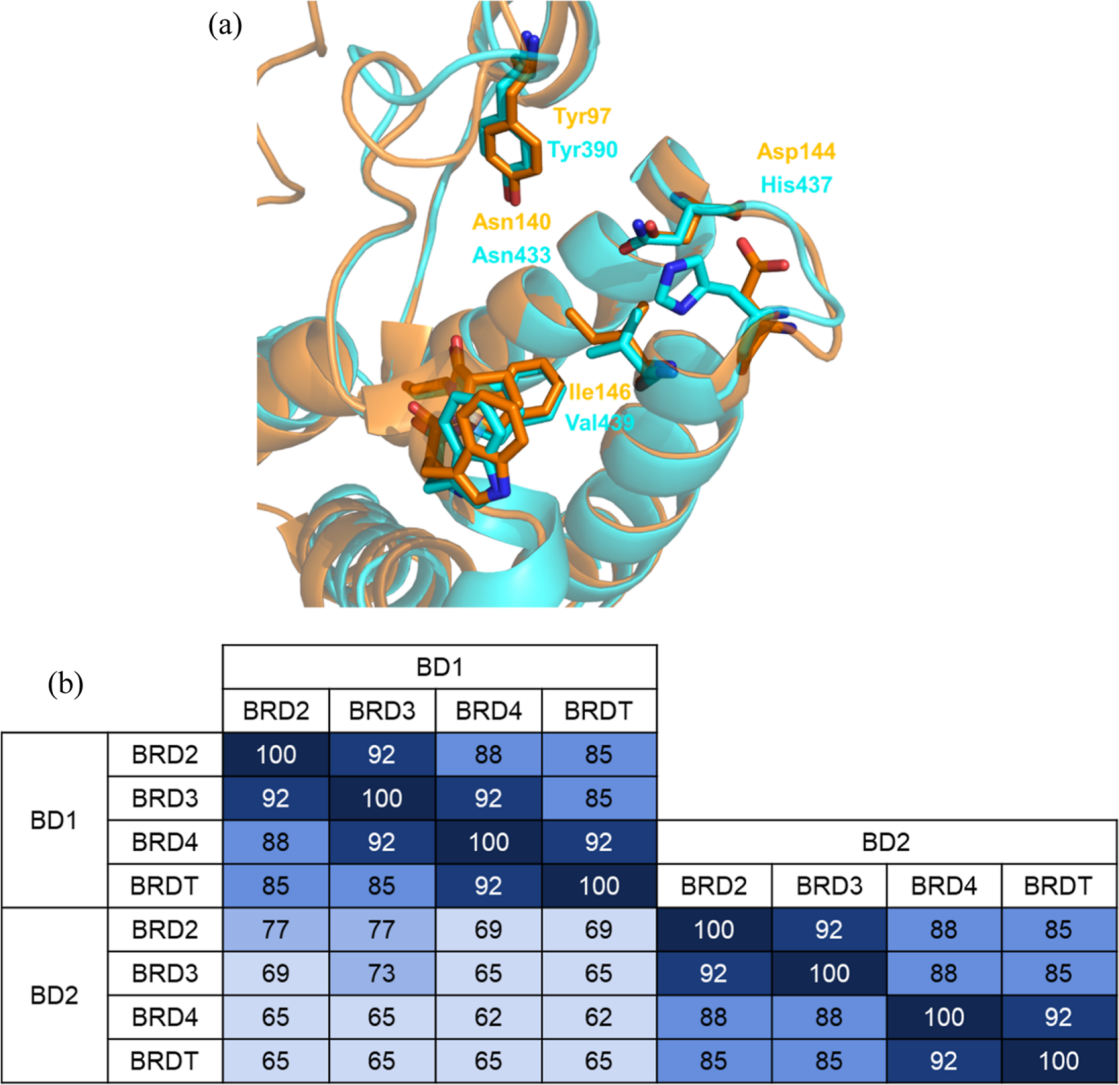
(a) Overlay of apo BRD4 BD1 in orange (pdb: 2oss) and apo BRD4 BD2 in cyan (pdb: 2ouo). Waters and ethylene glycol have been removed for clarity; (b) percent identity within the KAc binding site between the BET family bromodomains.^13^ The darker the shade of blue, the higher the bromodomain identify.

The 61 human bromodomains are a highly conserved 110 amino-acid structural motif found in 46 epigenetic bromodomain-containing proteins.^1^ These reader domains regulate gene expression through the recognition of histone tail-acetylated lysine (KAc) residues, with the resulting interaction initiating the recruitment of transcriptional regulatory proteins.^2^ Dysregulation of this critical epigenetic control mechanism drives profound biological phenotypes with bromodomain-containing proteins implicated in a variety of oncology and inflammatory disease states.^3,4^ Initially considered intractable,^5^ academic and industrial research into bromodomains has been accelerated through several seminal publications which demonstrated that the histone tail KAc–bromodomain protein–protein interaction was ligandable and could be disrupted by small molecule binding.^6−8^ Aided by the disclosure of high-quality chemical probes in 2010 and due to the profound disease-relevant biology associated with their dysregulation, the bromodomain and extra-terminal (BET) family of bromodomain-containing proteins have received a substantial research focus.^9−11^ The BET family is made up of four proteins, the ubiquitously expressed BRD2, BRD3, and BRD4, and the testes-restricted BRDT.^1^ Each protein contains tandem N-terminal bromodomains, termed BD1 (closer to the N-terminus) and BD2 (closer to the C-terminus), making a total of eight bromodomains in the BET family. Focusing on the KAc binding site, the BET BD1 bromodomains have a high degree of homology with each other, as do the BET BD2 bromodomains (85–92% sequence identity, Figure 1b).^12^ In contrast, the BET BD1 and BET BD2 domains are more different from each other, with 62–77% sequence identity. Overlaying apo structures for BRD4 BD1 (pdb: 2oss) and BRD4 BD2 (pdb: 2ouo) reveals some key amino acid residue differences between the two KAc binding sites (Figure 1a). Both bromodomains possess conserved Asn and Tyr residues, which make critical H-bonding interactions with KAc, or a small molecule mimetic. A tryptophan, proline, and phenylalanine (WPF) triad is present in both domains and forms a lipophilic shelf, which is accessed *via* a gatekeeper residue, Ile146 in BD1 and Val439 in BD2. Focusing on the key amino acid differences between the BD1 and BD2 KAc binding sites highlights acidic Asp144 in BRD4 BD1, where the same amino acid in three-dimensional space is replaced with basic His437 in BRD4 BD2. It is of note that this His is conserved in all of the BET BD2 bromodomains and swapped with Asp in all of the BET BD1 bromodomains. Analysis of the crystal structures shows that the BET BD1 Asp residues typically occupy a *gauche*^+^ chi1 rotation, which points the residue away from the KAc binding site. In contrast, the BET BD2 His residues are positioned toward the KAc binding site, adopting a *trans* chi1 conformation.

**Figure 2:**
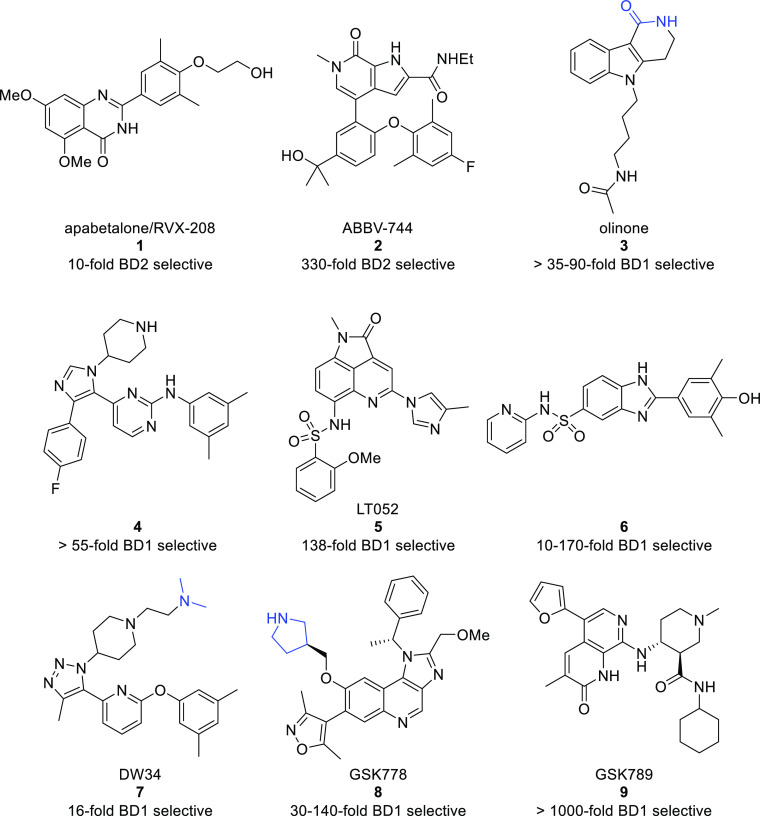
Structures of selected domain-biased/selective BET BD1 and BD2 bromodomain inhibitors. Where reported, the part of the molecules that drives BET BD1 domain selectivity through interacting with Asp144 (BRD4 BD1 numbering) is colored blue for clarity.

Small molecules which bind equipotently to all eight BET family bromodomains, typically in a 2:1 stoichiometry, are termed pan-BET inhibitors and have demonstrated efficacy not only in preclinical models of oncology and inflammation but also in oncology clinical trials.^14^ However, dose-limiting toxicity, including thrombocytopenia and gastro-intestinal findings, has been observed both preclinically and in clinical trials, which may ultimately limit the eventual therapeutic impact of this mechanism of action.^15,16^ To try and circumvent these toxicities and potentially expand beyond the clinical therapeutic potential of pan-BET bromodomain inhibitors, there has been an increasing focus on identifying domain-selective molecules that bind to either BD1 or BD2 of the BET family.^17,18^ These efforts have led to the disclosure of multiple structurally distinct and some highly BET domain-selective molecules from research efforts across academia and industry (Figure 2). Apabetalone/RVX-208 (**1**), the first reported BET BD2 biased compound, demonstrates a 10-fold selectivity over BET BD1.^19^ This molecule has advanced into multiple clinical trials for major adverse cardiac events and, more recently, for Covid-19 treatment.^20^ With a much improved 330-fold selectivity over BET BD1, ABBV-744 (**2**) became the first highly selective BET BD2 molecule to progress into clinical trials, although the phase I study in acute myeloid leukemia was terminated for strategic reasons.^21,22^

In contrast to BET BD2 domain-selective inhibitors, there have been fewer BET BD1 molecules reported, and none have yet entered clinical trials. Early disclosures of weakly active BET BD1 domain-selective molecules, such as olinone (**3**) (BRD4 BD1: *K*_d_: 3.4 μM), demonstrated an important proof of concept for BD1 domain selectivity with >35–90-fold selectivity over BD2 determined by isothermal titration calorimetry.^23^ The selectivity observed with olinone is proposed to derive from a steric clash with His437 in BD2 and a direct hydrogen bond with the corresponding Asp144 in BD1.^24^ Trisubstituted imidazole **4** is a dual p38a and BRD4 BD1 inhibitor with micromolar potency for BRD4 BD1 (IC_50_: 1.8 μM) and >55-fold selectivity over BRD4 BD2.^25^ The selectivity for BD1 over BD2 is proposed to originate, in part, from the fluorophenyl ring, displacing structurally conserved water from BD1 but not BD2. With an improved level of potency, tricyclic LT052 (**5**) displays an IC_50_ of 88 nM and 138-fold selectivity for BRD4 BD1 over BRD4 BD2. This level of selectivity is proposed to derive from a steric clash between the appended methyl imidazole group and His437 of BRD4 BD2. Taking an isosteric approach to replace a metabolically unstable azobenzene core, benzimidazole **6** bearing a phenol KAc mimetic demonstrates a BRD4 BD1 IC_50_ of 0.25 μM with 10–170-fold selectivity for BET BD1 over BD2.^26^ DW34 (**7**) has a methyl triazole group as the KAc mimetic and demonstrates a *K*_d_ of 12 nM with 16-fold selectivity for BRD4 BD1 over BRD4 BD2.^27^ Critical to driving this domain selectivity is a charge-assisted, through water, hydrogen bond between the dimethylamine group and the BD1-conserved Asp144 (Figure 1a). GSK778 (**8**), derived from pan-BET inhibitor I-BET151,^28^ has a BET BD1 IC_50_ of <100 nM and 30–140-fold selectivity over BET BD2.^29^ Like DW34 (**7**), GSK778 (**8**) targets the key amino acid swap of Asp144 in BD1 and His437 in BD2 with the pendant chiral pyrrolidine, making a through-water hydrogen bond to Asp144. Demonstrating a step-change in BET domain selectivity, GSK789 (**9**) is ≥ 1000-fold selective for the BET BD1 bromodomains with a BRD4 BD1 IC_50_ of 32 nM.^12^ The high levels of selectivity are thought to arise from a precise placement of the 2-furyl group in the narrow cleft between Leu92 and Trp81 (BRD4 BD1 numbering), referred to as the ZA channel, as well as a clash between the pendant amide and the BD2 conserved His residue. Additionally, demonstrating an important proof of concept for binding to a single BET bromodomain, compounds with selectivity for BRD4 BD1 over the other seven members of the BET bromodomain family have also been disclosed.^30−32^ As part of GSK’s wide-ranging bromodomain portfolio research strategy, multiple parallel studies were initiated to deliver high-quality domain-selective chemical probes and enable biological evaluation and dissection of the BET BD1 pharmacology, some of which have already been published.^12,28^ Of particular importance to these research efforts was the delivery of chemical probes with high potency, BET BD1 domain selectivity, and structural diversity to give increased confidence that any observed pharmacology could be attributed to the target.^33–35^ As part of this portfolio research strategy, herein, we describe a structure-guided design approach which identified and optimized a lipophilic efficient BET BD1-biased benzimidazole substructure into the high-quality BET BD1 domain-selective chemical probe GSK023.

## Results and Discussion

### Target Product Profile

The utility of a chemical probe in target validation is directly related to its quality, with important publications detailing requirements to aid researchers.^36−38^ Taking inspiration from this, the BET BD1 chemical probe requirements were defined as follows: Structural differentiation to internally developed (GSK778 (**8**) and GSK789 (**9**)) and reported BET BD1 chemical series (Figure 2) was critical. Activity against BET BD1 as judged by a TR-FRET assay pIC_50_ > 7.5 was required, together with an aspirational target of 1000-fold selectivity over BET BD2. It was accepted that selectivity would be judged initially through a mutant BRD4 TR-FRET assay and would likely shift depending on the protein construct and assay format. As for any chemical probe, proof of cellular target engagement was critical, and a target pIC_50_ > 7.0 in a lipopolysaccharide (LPS)-stimulated human whole blood (hWB) assay measuring release of the proinflammatory cytokine monocyte chemoattractant protein-1 (MCP-1) was set. Previous studies have shown that BET BD1 inhibition is sufficient to inhibit *in vitro* production of MCP-1 in LPS-stimulated hWB and, as such, served as a relevant measure of cellular target engagement and, concomitantly, proof of cellular permeability.^12,39^ Selectivity over other human bromodomains outside of the BET family, known collectively as non-BET bromodomains, was also required,^40^ with *a* ≥ 100-fold selectivity window targeted. To ensure the molecules remained within developable chemical space, a target property forecast index (PFI) as defined by chromLog*D*_pH7.4_ + number of aromatic rings ≤6 was set.^41^ Finally, for any high-quality chemical probe, sufficient aqueous solubility is critical, so a solubility of >100 μg/mL was required.

### Hit Identification

**Figure 3:**
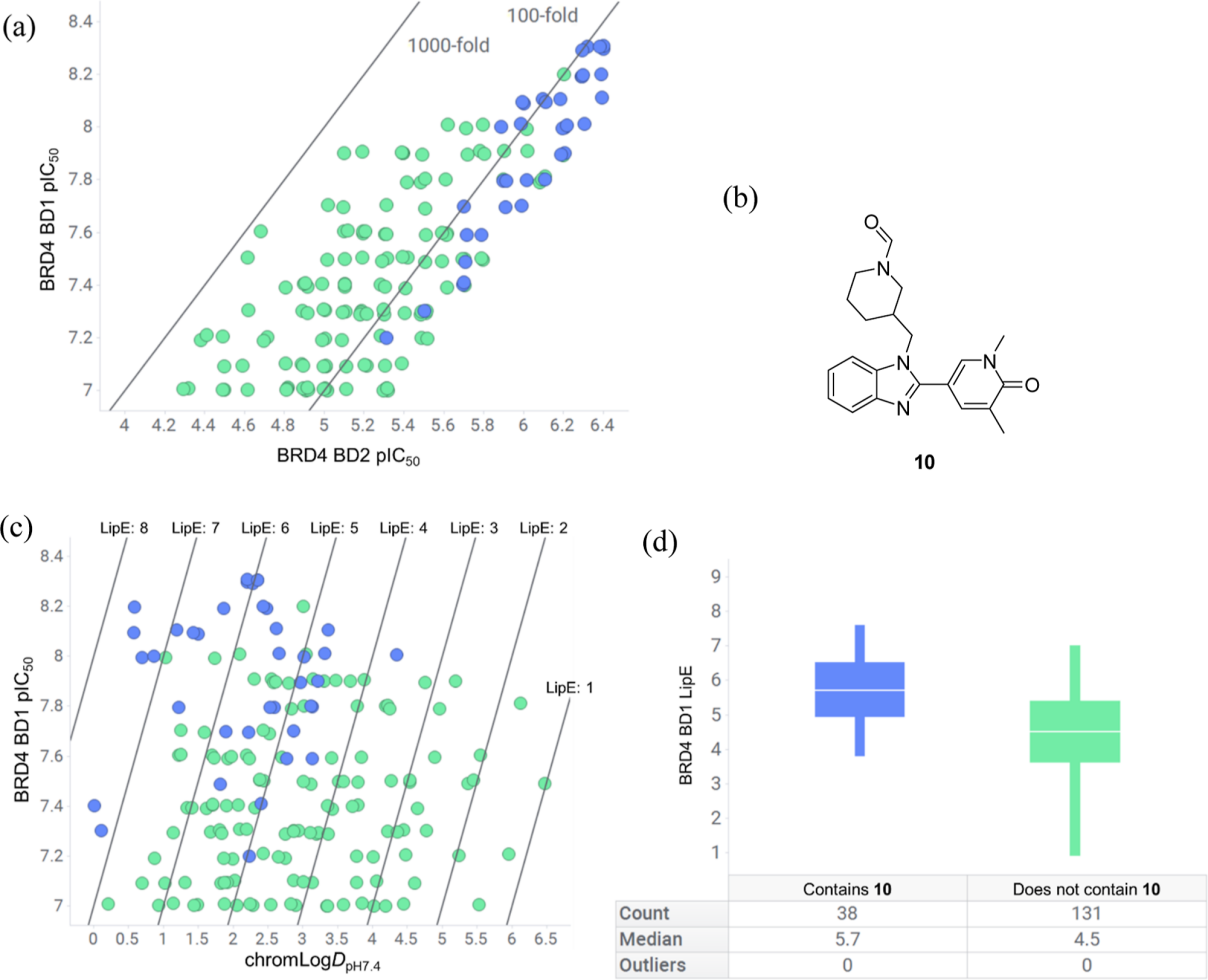
(a) Plot of BRD4 BD1 pIC_50_ against BRD4 BD2 pIC_50_ for 169 compounds with BRD4 BD1 pIC_50_ ≥ 7 and BRD4 BD1 domain selectivity ≥50-fold, with diagonal lines representing BD1 domain selectivity. Compounds containing substructure **10** are colored blue, and those that do not contain substructure **10** are colored green; (b) substructure **10**; (c) plot of BRD4 BD1 pIC_50_ against chromLog*D*_pH7.4_ with diagonal lines representing different LipE values for 169 molecules. Compounds containing substructure **10** are colored blue, and those that do not contain substructure **10** are colored green; (d) comparison of median LipE values for compounds containing substructure **10** and those that do not.

As a strategic decision, every compound synthesized across all of GSK’s bromodomain research projects was tested in both a BRD4 BD1 and a BRD4 BD2 TR-FRET assay as part of tier 1 data generation. These robust domain-selective assays not only had a high weekly capacity but also served as representative members of the rest of the BET bromodomain family. This BRD4 BD1/BD2 domain selectivity data, combined with the output of several historical diversity and knowledge-based screening campaigns, provided a rich data set from which to identify suitable hits/series to deliver a BET BD1 chemical probe. With BRD4 BD1 and BD2 full curve data on ∼25,000 active compounds, all compounds with a BRD4 BD1 pIC_50_ < 7 were filtered out. Compounds with ≥50-fold BRD4 BD1 selectivity over BRD4 BD2 from chemotypes not previously prosecuted internally or known in the literature were prioritized to give 169 compounds a manageable number for visual inspection. Plotting the compounds with BRD4 BD1 pIC_50_ on the *y*-axis and BRD4 BD2 pIC_50_ on the *x*-axis allowed visualization of selectivity for the compounds, with a range of domain selectivity from 50-fold up to ∼800-fold (Figure 3a). A series of 38 benzimidazole compounds with substructure **10** was noticeable among the structures, although these compounds were at the lower end (50–126-fold) of BD1 domain selectivity. The chromatographic Log*D* at pH 7.4 (chromLog*D*_pH7.4_) had also been routinely measured, and plotting BRD4 BD1 pIC_50_ on the *y*-axis and lipophilicity on the *x*-axis facilitated analysis of the lipophilic efficiency (LipE) of the compound set (Figure 3c).^42^ LipE is a straightforward, yet powerful metric for medicinal chemistry teams to normalize potency relative to lipophilicity, a critical factor both for parameters of the human dose equation and also for promiscuity.^43,44^ To aid interpretation, diagonal lines representing different LipE values are shown, with the most desirable space found toward the top left-hand area of the plot representing molecules with the comparatively highest potency per unit of lipophilicity.

The lipophilic efficiency analysis provided a critical insight into compound quality, with the 38 compounds containing benzimidazole substructure **10** among the most lipophilic efficient in the data set. Analysis revealed that the piperidine-substituted benzimidazole **10** had a median LipE of 5.7 in contrast to those compounds that did not contain substructure **10**, which had a far reduced median LipE of 4.5 (Figure 3d). The benzimidazole-containing compounds had been synthesized as part of a research effort to deliver pan-BET bromodomain inhibitors that culminated in the discovery of the high-quality oral candidate I-BET469.^45^ However, the clear trend of domain selectivity and LipE for substructure **10** was not apparent to us until these efforts to deliver a BET BD1 domain-selective chemical probe were initiated.

**Scheme 1:**
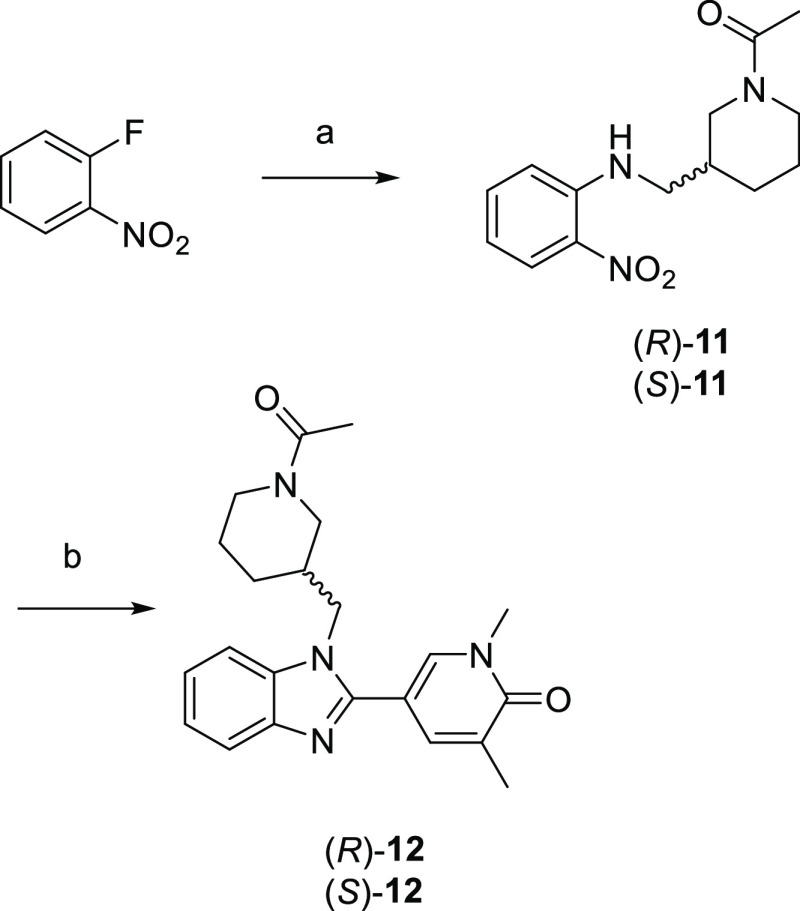
Synthesis of (*R*)-**12** and (*S*)-**12** Reagents and conditions: (a) (*R*)- or (*S*)-1-(3-(aminomethyl)piperidin-1-yl)ethan-1-one, DIPEA, NMP, 200 °C, 1 h; (b) 1,5-dimethyl-6-oxo-1,6-dihydropyridine-3-carbaldehyde, Na_2_S_2_O_4_, EtOH, H_2_O, 100 °C, 130 min.

Despite the comparatively lower levels of domain selectivity to other molecules in the data set (Figure 3a), the higher levels of lipophilic efficiency suggested that investigating the benzimidazole series further would be a promising approach to delivering a high-quality BD1 chemical probe meeting the target product profile (Figure 3c). To better understand the profile of benzimidazole substructure **10**, the individual enantiomers of acetylated piperidine **12** were accessed (Scheme 1). Facile base-mediated SNAr with the appropriate commercially available enantiopure acetylated piperidine provided access to nitro intermediates **11**. A subsequent one-pot reduction-cyclization sequence with sodium dithionite and the appropriate pyridone aldehyde provided the individual enantiomers of **12**.^44^

Profiling the individual enantiomers (*R*)-**12** and (*S*)-**12** revealed a modest 3-fold eudysmic ratio at BRD4 BD1 (Table 1). Activity at BRD4 BD2 remained much the same for both compounds, but as the *S*-enantiomer demonstrated 10 nM potency at BRD4 BD1 (pIC_50_: 8.0), this translated into 63-fold selectivity for BRD4 BD1. This domain selectivity window was combined with highly encouraging ligand efficiency (LE) and LipE, as well as high aqueous kinetic solubility, as judged by a chemiluminescent nitrogen detection (CLND) assay. A chromLog*D*_pH7.4_: 2.2 and three aromatic rings meant (*S*)-**12** had a PFI 5.2, within the target criteria ≤6. Highlighting the importance of the chiral acetylated piperidine for domain selectivity, previously disclosed matched molecular pair tetrahydropyran **13** only showed 6-fold BRD4 BD1 selectivity due to substantially lower activity at BD1 and slightly higher activity at BD2.^44^

**Table 1:**
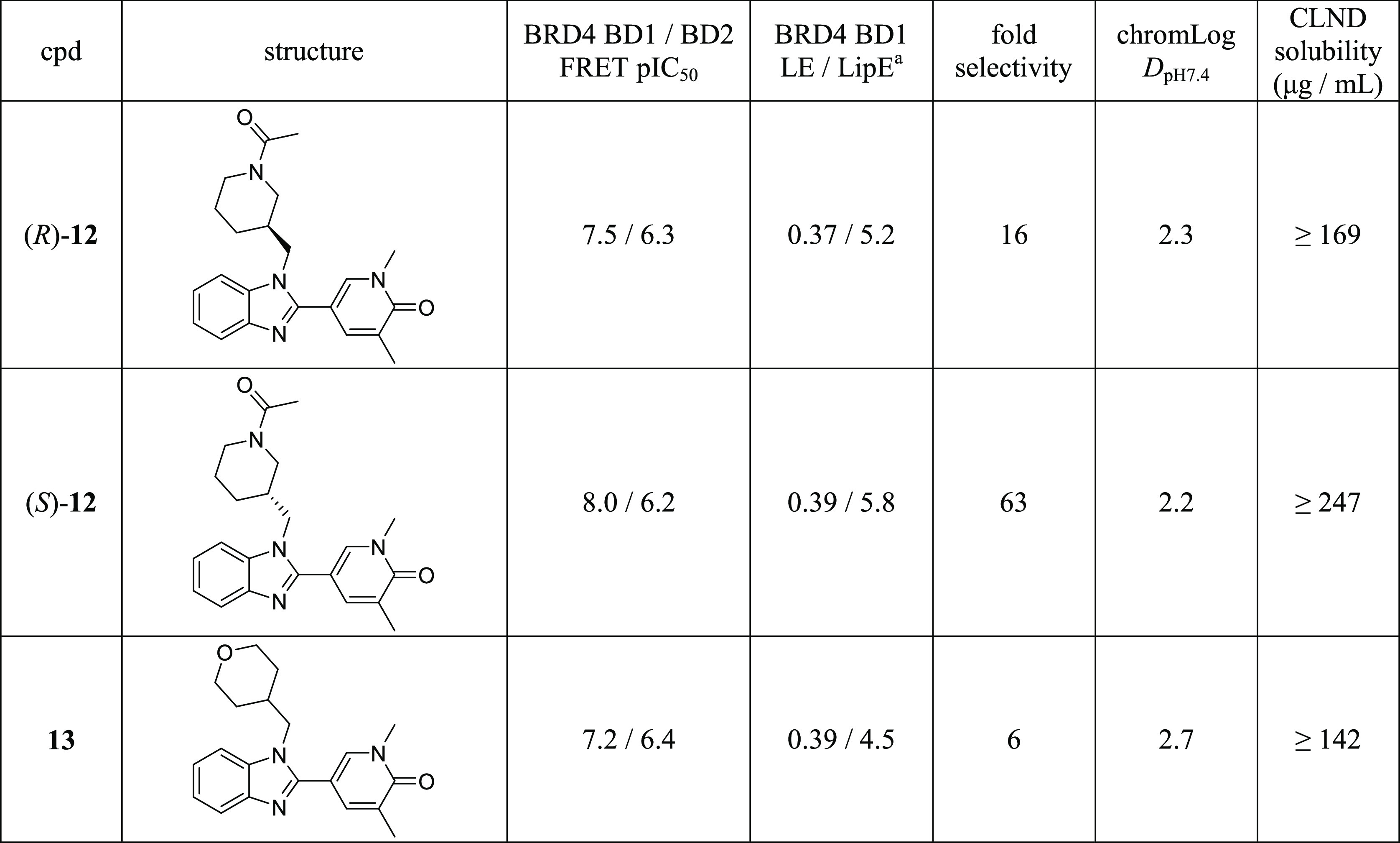
Profile of (*S*)-**12** and (*R*)-**12**

a LipE = BRD4 BD1 pIC_50_–chromLog*D*_pH7.4_; LE = (1.37 × BRD4 BD1 pIC_50_)/heavy atom count.

**Figure 4:**
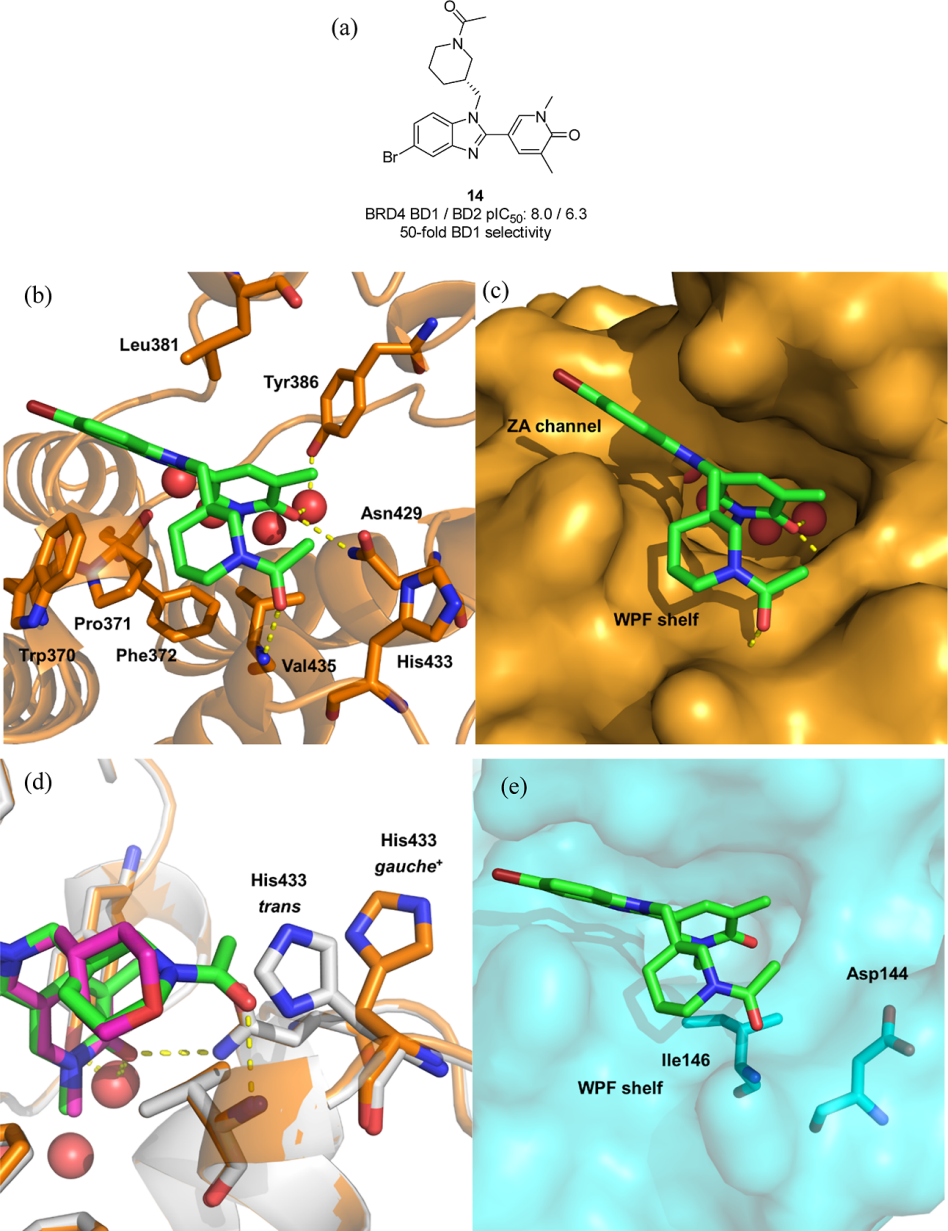
(a) Structure of **14**; (b) crystal structure of **14** (green) bound to BRD2 BD2 (orange) (pdb: 8px8). Water molecules are shown as red spheres and hydrogen bonds are marked in yellow; (c) As (b), but with the protein surface shown in orange; (d) overlay of the crystal structure of **13** (pink) bound to BRD2 BD2 (gray) (pdb: 8px2) and the crystal structure of **14** (green) bound to BRD2 BD2 (orange) (pdb: 8px8); (e) As (b) but with an overlay of the BRD4 BD1 protein surface (cyan) bound to **13** (pdb: 6tpy).

To shed light on the origin of the domain selectivity, we took advantage of the presence of a historical internal crystal structure of **14**, a close analogue of (*S*)-**12** bearing a bromo-substituent in the C-5 position of the benzimidazole, bound to BRD2 BD2 (Figure 4).

Benzimidazole **14** demonstrates 50-fold BD1 domain selectivity, comparable to the 63-fold observed for (*S*)-**12**, and served as a useful surrogate to understand the binding mode and potential reasons for the observed domain selectivity. As expected, the dimethyl pyridone group functions as the KAc mimetic, making the canonical hydrogen bonding interaction to Asn429 and through water to Tyr386 (BRD2 BD2 numbering). The flat benzimidazole ring is positioned in the narrow ZA channel, sandwiched between Leu381 and Trp370, with the bromine pointing into the solvent (Figure 4c). The appended piperidine ring sits on the lipophilic WPF shelf with the amide carbonyl, making a direct 2.8 Å hydrogen bond with the backbone NH of gatekeeper Val435. Interestingly, His433 sits in a *gauche*^+^ conformation, in contrast to the *trans* conformations found in the BET BD2 apo structures (Figure 1a). The crystal structure of the nondomain-selective benzimidazole **13** bound to BRD2 BD2 had been previously solved in GSK, and comparison proved enlightening (Figure 4d). The two molecules overlay almost perfectly, apart from the WPF shelf region. The tetrahydropyran group does not interact with gatekeeper Val435, and His433 is in the same *trans* conformation, as observed in the apo structure. The acetyl group of **14** would sterically clash with His433 if the side chain was in a *trans* conformation, which presumably forces a conformer change to *gauche*^+^ to accommodate. Overlaying the apo BRD4 BD1 surface shows how Asp144 already sits in a *gauche*^+^ conformation pointing away from the KAc binding site and would accommodate **14** without requiring any protein rearrangement (Figure 4e). The hydrogen bond between the piperidine amide carbonyl and backbone NH is expected to be maintained with BRD4 BD1, in this case with gatekeeper residue Ile146, the amino acid analogous to Val435 in BRD2 BD2. As such, the working hypothesis at this stage of the project was that the encouraging levels of selectivity between BD1 and BD2 for (*S*)-**12** was due to a steric clash with the apo BD2 *trans* conformation His433, which necessitated a high-energy protein rearrangement to bind.

**Figure 5:**
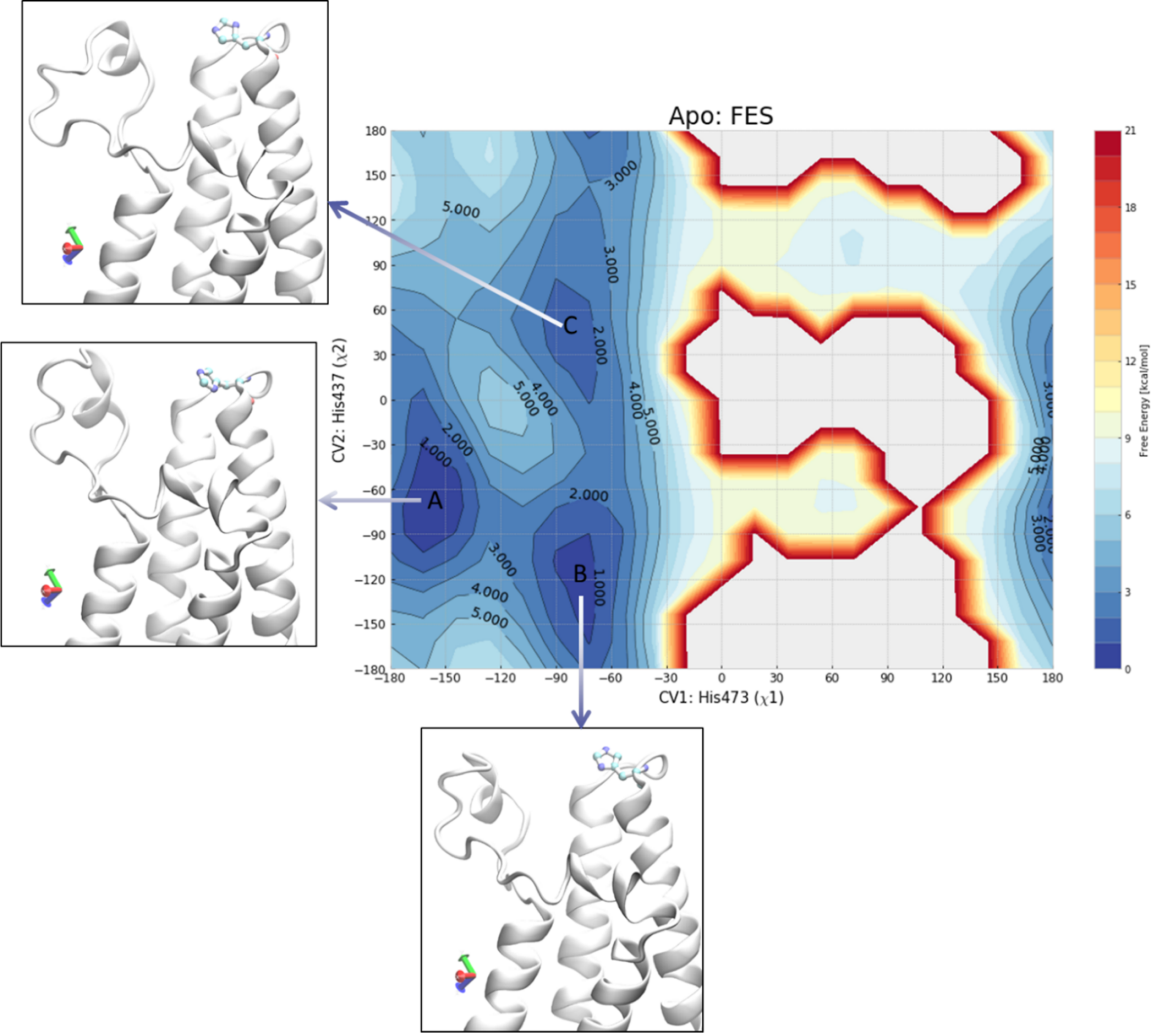
Two-dimensional plot of the average conformational FES of apo BRD4 BD2 (pdb: 2ouo) as calculated by metadynamics. In the boxes, representative conformations of the minima A (His437 *trans*), B (His437 *gauche*^+^), and C (His437 *gauche*^+^) of the apo structure are reported. The free energy is displayed every 1 kcal/mol, and the contour levels are shown up to 5 kcal/mol.

To evaluate this hypothesis further, metadynamics simulations were setup for apo BRD4 BD2 (pdb: 2ouo).^46,47^ Metadynamics is an enhanced sampling simulation that uses a history-based biasing potential to encourage the simulation to cross high energy barriers and visit otherwise poorly sampled states. The biasing potential is constructed as a sum of repulsive Gaussians centered along the trajectory of collective variables (CV), and, at the end of the simulation, the free energy surface (FES) can be calculated. To explore a potential energy penalty for rearrangement of BRD4 BD2 His437, the collective variables for the simulation were the chi1 and chi2 of His437. The resulting two-dimensional FES showed the presence of three distinct basins corresponding to specific His437 conformations (Figure 5). Interestingly, and in contrast to our hypothesis, A (His437 *trans*), B (His437 *gauche*^+^), and C (His437 *gauche*^+^) are all predicted to have comparable free energy and, as such, are expected to be similarly populated in solution. This simulation suggested that BRD4 BD2 His437 can readily access both *gauche*^+^ and *trans* conformation in the apo form and that the conformer change upon binding to (*S*)-**12** would not be expected to automatically drive domain selectivity for BET BD1 over BET BD2.

### Hit Optimization

Despite not being able to fully explain the domain selectivity of (*S*)-**12**, this hit represented a promising starting point from which to further improve the domain selectivity window and deliver a BET BD1 chemical probe. As previously discussed, across the BET bromodomains, there is a His/Asp switch between BD2 and BD1 (Figures 1 and 4). We sought to target Asp144 (BRD4 BD1) with a basic group to not only gain potency at BD1, but also clash with the basic His437 found in the BD2 bromodomains. As the metadynamics simulation indicated that there was little energy penalty for BRD4 BD2 His437 rearranging from *trans* to *gauche*^+^, we looked to ensure repulsion with His437 in the *gauche*^+^ position to drive further selectivity. Inspection of the overlay of **14** and BRD4 BD1 revealed that Asp144 was 5 Å from the acetate methyl, and it was hypothesized that a linker of at least two carbon atoms followed by a hydrogen-bond donor would likely be required to make an interaction (Figure 4e). From a visual inspection of the benzimidazole-containing compounds made historically at GSK, it was considered that none of the compounds were capable of making such an interaction. Experience gained within GSK from the delivery of I-BET469 from the same benzimidazole template drove a strategic choice to focus on elaboration of the piperidine motif at the expense of further benzimidazole ring substitution as the highest probability of success approach to deliver a BET BD1 chemical probe.^44^

To achieve this goal, a flexible synthetic intermediate was sought that would allow the introduction of late-stage diversity *via* array chemistry.

**Scheme 2:**
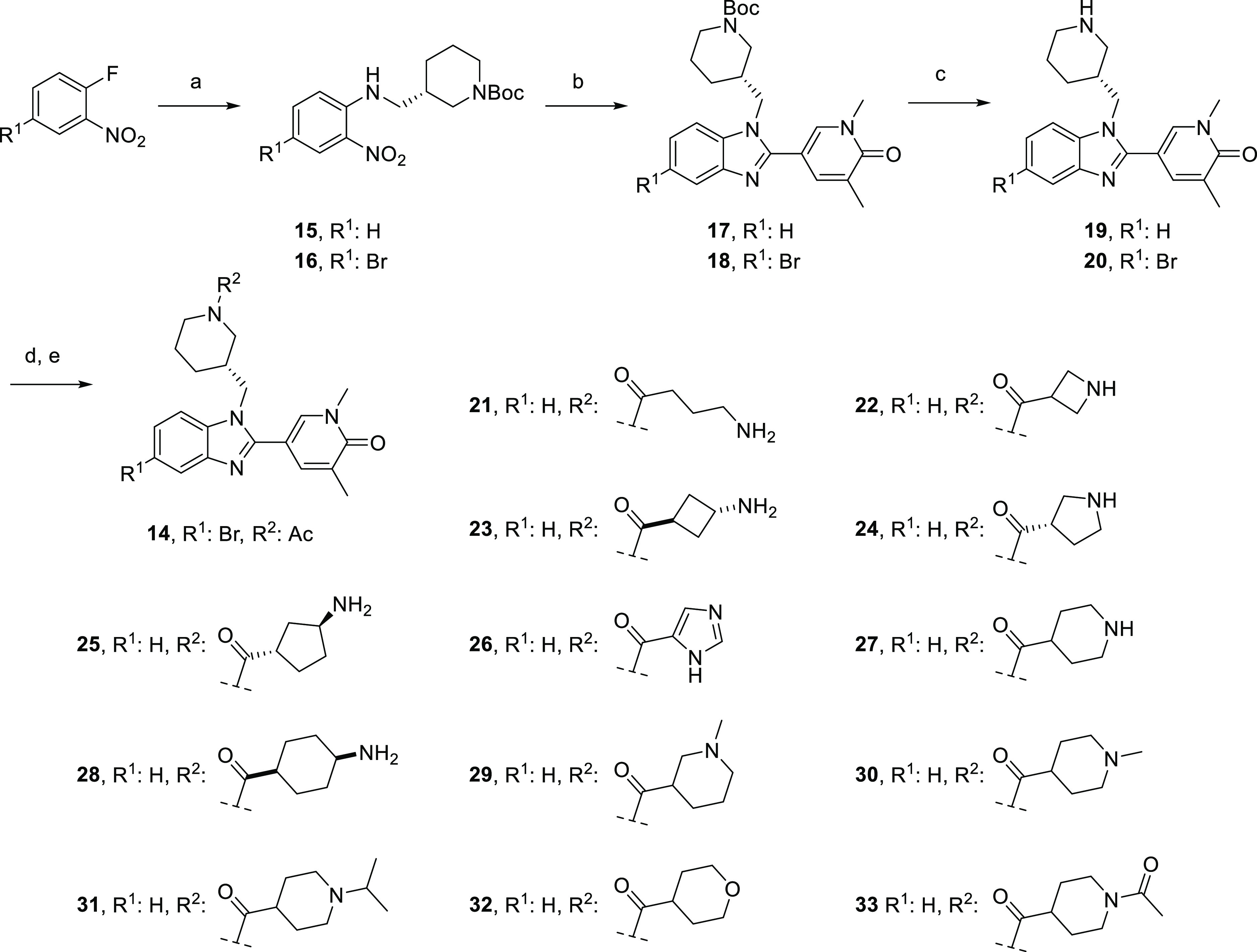
Synthesis of Benzimidazoles **14** and **21**–**33** Reagents and conditions: (a) *tert*-butyl (*S*)-3-(aminomethyl)piperidine-1-carboxylate, K_2_CO_3_, DMF, 80–100 °C; (b) 1,5-dimethyl-6-oxo-1,6-dihydropyridine-3-carbaldehyde, Na_2_S_2_O_4_, EtOH, H_2_O, 100 °C; (c) HCl, 1,4-dioxane, 0 °C-rt; (d) acid, HATU, DIPEA, DMF, rt or acid chloride, NEt_3_, THF, rt; (e) For **21**–**25**, **27**–**29**: HCl, 1,4-dioxane, rt.

As in Scheme 1, SNAr between 1-fluoro-2-nitrobenzene and the commercially available Boc-protected enantiopure (*S*)-amino piperidine building block provided aryl nitro intermediate **15** (Scheme 2). Subsequent sodium dithionate-mediated reduction-cyclization with the appropriate pyridone aldehyde gave Boc-protected benzimidazole **17**. Acid-mediated deprotection provided key intermediate **19**, which was smoothly converted into targets **21**–**33***via* amide coupling, followed by acid-mediated Boc deprotection (if required). Historical 5-bromo **14** was accessed *via* an analogous sequence starting with 4-bromo-1-fluoro-2-nitrobenzene instead of 1-fluoro-2-nitrobenzene.

**Scheme 3:**
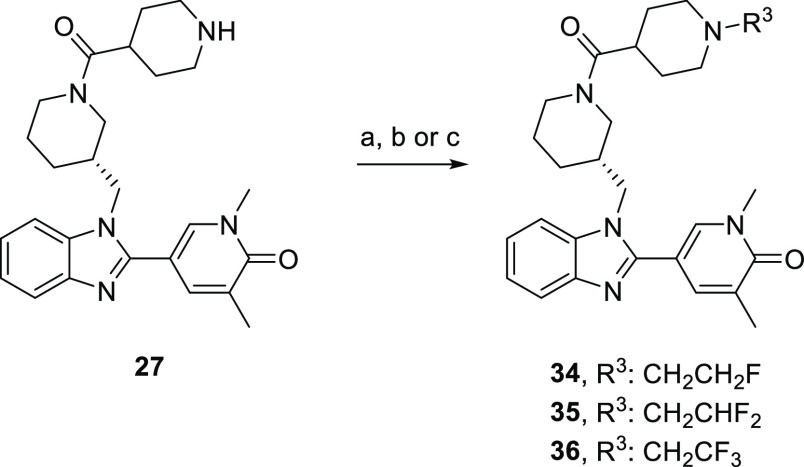
Synthesis of Benzimidazoles **34**–**36** Reagents and conditions: (a) For **34**: BrCH_2_CH_2_F, NaH, DMF, rt to 60 °C, 1 h, 37%; (b) For **35**: BrCH_2_CHF_2_, NaH, DMF, rt to 90 °C, 37 h, 37%; (c) For **36**: TFA, PhSiH_3_, THF, 70 °C, 19 h, 44%.

To access fluorinated analogues **34**–**36**, NH piperidine **27** was further derivatized by *N*-alkylation with the appropriate alkyl bromide or by reductive coupling with TFA and phenylsilane (Scheme 3).

Comparing the profiles of acetyl-substituted (*S*)-**12** and synthetic intermediate **19** bearing an unsubstituted piperidine ring demonstrated the importance of the acetyl group and/or ablating basicity in driving not just BRD4 BD1 potency but also selectivity over BRD4 BD2 (Table 2). Growing the acetyl group to give **21** with a flexible aminopropyl moiety confirmed the design hypothesis that a pendant basic group would drive improved domain selectivity, with 158-fold BRD4 BD1 selectivity obtained. However, the improvement in domain selectivity was achieved entirely through reducing BRD4 BD2 potency, whereas it had been hypothesized that a basic group from this vector would also drive BRD4 BD1 potency *via* a specific salt bridge with Asp144. Nonetheless, we were highly encouraged by the improved BRD4 BD1 domain selectivity window over BRD4 BD2, which gave us reason to believe that this approach would ultimately deliver the target product profile. Introduction of the basic amine also reduced lipophilicity by one log unit and concomitantly improved BRD4 BD1 LipE 6-fold. To understand cellular target engagement, **21** was tested in an LPS-stimulated hWB assay measuring inhibition of the inflammatory mediator MCP-1 secretion. 500 nM activity was seen in this assay, confirming engagement with the BET bromodomains; however, the hWB activity represented a 40-fold drop when compared to the BRD4 BD1 potency, which was attributed to the pendant basic amine (predicted p*K*_a_: 10) driving poor passive permeability. As expected, high levels of aqueous solubility were retained with **21**, as determined by a high-throughput charged aerosol detection (CAD) method which replaced the previous CLND solubility assay at GSK. Across a large set of compounds from a variety of chemotypes, the aqueous solubility data was comparable between the CAD and CLND assay formats.

**Table 2:**
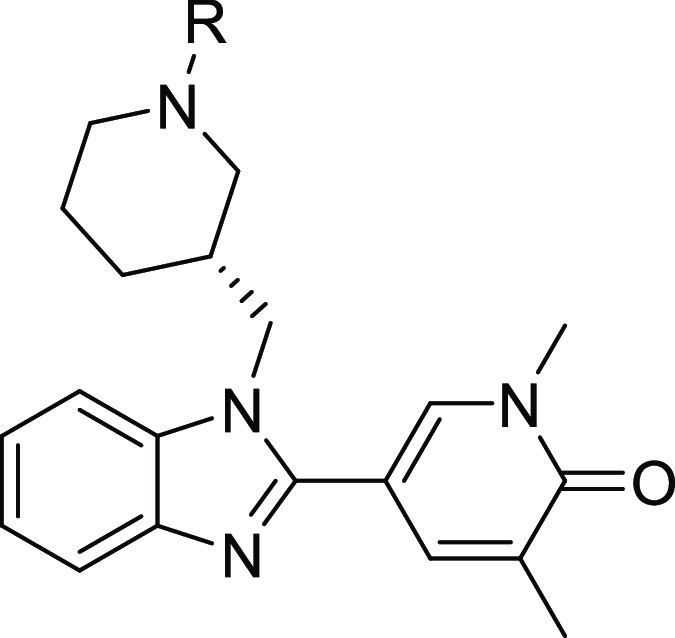

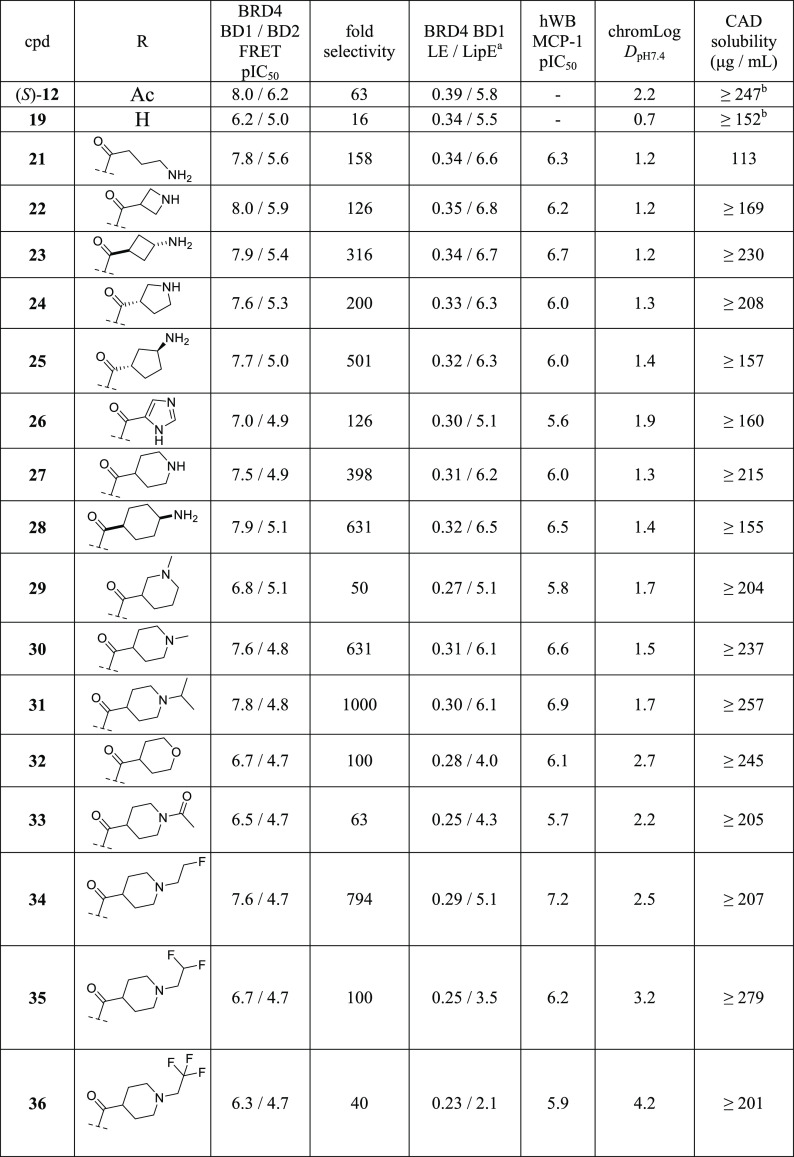
Profile of Domain-Selective Benzimidazoles (*S*)-**12**, **19**, **21–36**

a LipE = BRD4 BD1 pIC_50_–chromLog*D*_pH7.4_; LE = (1.37 × BRD4 BD1 pIC_50_)/heavy atom count.

b CLND solubility.

Constraining the alkyl chain with azetidine **22**, *trans*-substituted cyclobutane **23**, or (*S*)-pyrrolidine **24** had minimal impact on the BRD4 BD1/BD2 potency, domain selectivity, or physicochemical properties, although **23** demonstrated improved hWB MCP-1 activity relative to **21**. Substituted (*S*,*S*)-cyclopentane **25** showed an encouraging improvement in domain selectivity, with 500-fold BD1 selectivity being obtained for the first time from the benzimidazole series. As with propylamine **21**, this domain selectivity was due to a decrease in BRD4 BD2 activity rather than any increase in BRD4 BD1 potency. The hWB MCP-1 activity demonstrated cellular target engagement, albeit with a substantial drop-off between the biochemical and cellular potency. Imidazole **26** was not well tolerated, with a 10-fold drop in BRD4 BD1 potency relative to acetate (*S*)-**12**. Appending a 6-membered ring, as in either piperidine **27** or *trans*-cyclohexyl **28**, showed encouraging levels of BD1 domain selectivity but was still short of the predetermined target of 1000-fold. Comparing 3-substituted racemic piperidine **29** with matched molecular pair 4-substituted analogue **30** highlighted the importance of the location of the amine in driving both BRD4 BD1 activity and BD1 domain selectivity over BRD4 BD2, with a drop in both parameters seen with 3-substituted piperidine **29**. This SAR strongly suggested that a specific interaction with the protein was occurring but did not explain why the BRD4 BD1 potency of **30** was still comparable to that of acetyl (*S*)-**12**, which lacked a basic group. *N*-Methylpiperidine **30** also had improved hWB MCP-1 activity compared to the NH-matched molecular pair **27** despite similar levels of BRD4 BD1 activity, which is presumably due to improved passive permeability driven by increased lipophilicity or a reduction in hydrogen-bond donors. Growing the methyl group of piperidine **30** to an iso-propyl group gave piperidine **31**, which demonstrated 1000-fold BD1 domain selectivity over BRD4 BD2 for the first time, together with hWB MCP-1 IC_50_: 126 nM (pIC_50_: 6.9) and excellent levels of aqueous solubility (≥257 μg/mL). Interestingly, neutral tetrahydropyran **32** and acetate **33** were less active at BRD4 BD1 without impacting activity at BRD4 BD2, which drove a much smaller selectivity window. Proximal fluorination is a common method to modulate the p*K*_a_ of basic amines and was used to test the hypothesis that basicity was required to drive both activity and selectivity for BRD4 BD1.^48^ Monofluoroethyl **34** (p*K*_a_: 7.3) showed minimal change in BRD4 BD1 potency when compared to *iso*-propyl **31** (p*K*_a_: 9.6), despite being 200-fold less basic and with ∼800-fold BD1 domain selectivity observed. Decreasing basicity further with difluoroethyl **35** (p*K*_a_: 4.2) had a substantial impact on BD1 potency and eroded the domain selectivity 100 fold. Neutral trifluoroethyl **36** further reduced the BRD4 BD1 activity with a concomitant drop in domain selectivity. Interestingly, the BRD4 BD2 activity of **30**–**36** remained essentially unchanged, with the dramatic changes in BD1 domain selectivity entirely due to changes in BRD4 BD1 potency.

### Understanding BD1 Domain Selectivity and Activity

**Figure 6:**
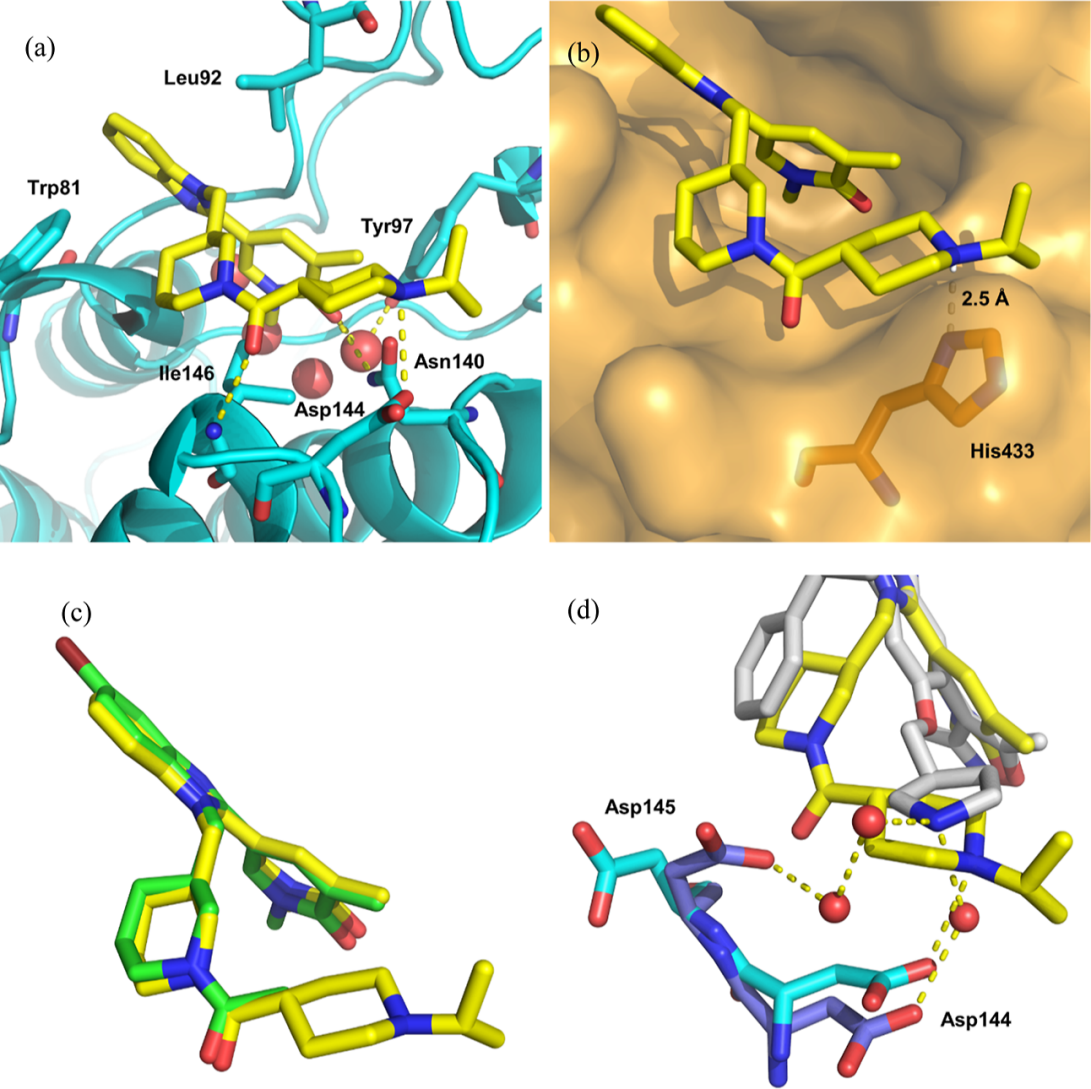
(a) Crystal structure of **31** (yellow) bound to BRD4 BD1 (cyan) (pdb: 8pxa). Water molecules are shown as red spheres and hydrogen bonds are marked in yellow; (b) As (a), but with the protein surface from **14** bound to BRD2 BD2 shown in orange overlaid (pdb: 8px8); (c) overlay of **31** (yellow) bound to BRD4 BD1 (pdb: 8pxa) with **14** (green) bound to BRD2 BD2 (pdb: 8px8); (d) overlay of **31** (yellow) bound to BRD4 BD1 (cyan) (pdb: 8pxa) and GSK778 (**8**) (gray) bound to BRD4 BD1 (blue) (pdb: 6swn). Only Asp145 and Asp144 are shown for clarity.

With piperidine **31** demonstrating an encouraging profile and 1000-fold BD1 domain selectivity, crystallography in complex with BRD4 BD1 was obtained (Figure 6).

As with benzimidazole **14**, the pyridone group of **31** acted as the KAc mimetic, making the predicted hydrogen-bonding interactions directly to Asn140 and through water to Tyr97 (Figure 6a). The benzimidazole core occupies the ZA channel formed between Trp81 and Leu92, with the latter amino acid making a 3.8 Å CH-π interaction with the imidazole centroid. As postulated for **14** bound to BD1 (Figure 4e), the pendant piperidine ring sits on the WPF shelf, with the carbonyl making a direct hydrogen bond to the backbone NH of gatekeeper Ile146. Demonstrating the design hypothesis, the basic piperidine (p*K*_a_: 9.6) interacts with Asp144 *via* a 3.0 Å salt bridge. Overlaying the protein surface from **14** bound to BRD2 BD2 indicates both a steric and electronic clash between the isopropyl-substituted piperidine and the *gauche*^+^-orientated imidazole ring of His433 in BD2 with the basic piperidine nitrogen and imidazole nitrogen only 2.5 Å apart (Figure 6b). Presumably, this clash between **31** and His433 contributes to the high levels of BD1 domain selectivity through the reduction of BD2 domain affinity. Comparing the bound conformation and orientation of **14** in complex with BRD2 BD2 and **31** bound to BRD4 BD1 shows almost no movement in extension from the piperidine acetyl group to make the salt bridge with Asp144 (Figure 6c). Interestingly, comparing the location of Asp144 between the BRD4 BD1 apo structure and that bound to **31** shows that only a slight 0.6 Å shift occurs to make this interaction with **31**. The direct salt bridge between the basic piperidine of **31** and Asp144 is unique among published BET BD1 domain-selective molecules, with other basic groups interacting with Asp144 by a through-water hydrogen bond (Figure 2).^49^ As an example, GSK778 (**8**) (Figure 2) makes a through-water hydrogen bond interaction from a pendant basic pyrrolidine group to Asp144 (Figure 6d).^28^ Interestingly, Asp145 also rotates toward GSK778 (**8**) to make an additional interaction with the pyrrolidine through two water molecules. In direct contrast, Asp145 remains in the same position as the apo BRD4 BD1 structure when bound to **31,** and a direct salt bridge is made with Asp144 by the basic piperidine.

The crystallography of **31** bound to BRD4 BD1 clearly showed a direct salt bridge with Asp144 (Figure 6a), an interaction that could not be present with (*S*)-**12**, which lacks a basic group in the appropriate region of the protein. While **31** is far more BD1 domain-selective than (*S*)-**12** due to decreases in BD2 activity (1000-fold compared to 63-fold, respectively), they both have comparable BRD4 BD1 potency (pIC_50_: 7.8 and 8.0, respectively), despite the additional salt bridge interaction that **31** makes. To probe this finding, the water network at the BRD4 BD1 KAc binding site was investigated with WaterMap calculations to compute the free energy of hydration (Δ*G*_hyd_) for water molecules from molecular dynamic simulations.^50,51^

**Figure 7:**
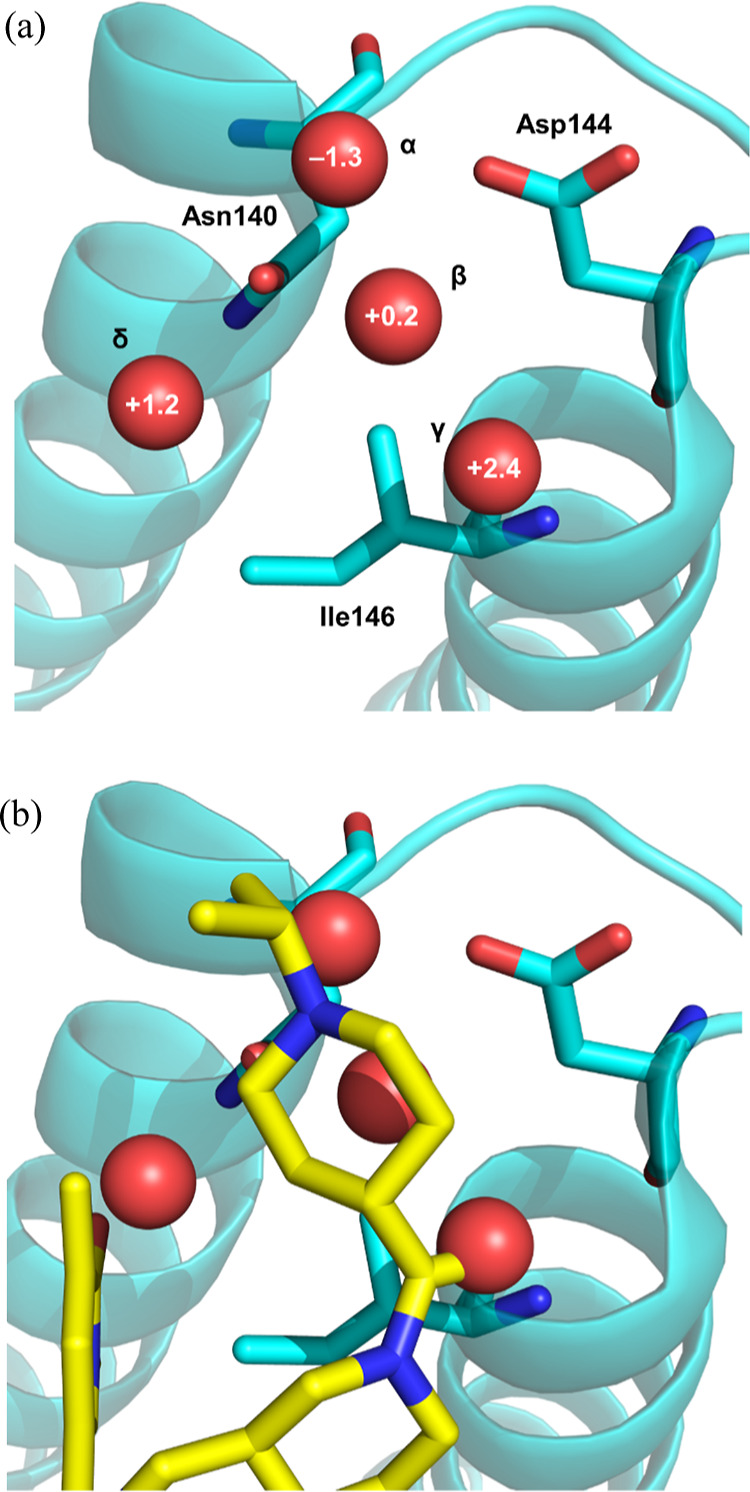
(a) Apo BRD4 BD1 (cyan) (pdb: 2oss). Waters are shown as red spheres, with respective Δ*G*_hyd_ in kcal/mol shown in white text; (b) as (a), but with **31** bound to BRD4 BD1 (yellow) overlaid (pdb: 8pxa).

WaterMap analysis of the apo BRD4 BD1 KAc binding site revealed the presence of several key hydration sites with differing free energies (Figure 7a). Water site γ is in proximity to the backbone NH of gatekeeper residue Ile146 and has a predicted Δ*G*_hyd_: +2.4 kcal/mol, suggesting this water site can be beneficially occupied by a small molecule. Water sites β and δ near Asn140 also have positive Δ*G*_hyd_ values, indicating they are energetically unfavorable, while, in direct contrast, water site α between Asp144 and the backbone NH of Lys141 is far more stabilized with a negative predicted Δ*G*_hyd_: −1.3 kcal/mol. Rerunning the WaterMap simulations after docking (*S*)-**12** into the apo BRD4 BD1 structure revealed that water site α becomes substantially more stabilized with a predicted Δ*G*_hyd_: −3.9 kcal/mol. Overlaying these water sites with the structure of **31** bound to BRD4 BD1 reveals that the compound must displace all of the water molecules and occupy these sites. The piperidine acetyl carbonyl displaces the energetically unfavorable water at water site γ to make a direct hydrogen bond with the Ile146 backbone NH, which presumably results in an affinity gain (Figure 7b). This is consistent with the +0.8 log unit potency difference between **13**, which does not interact with Ile146, and (*S*)-**12**, which can (Figure 4d). The pyridone KAc mimetic occupies a water site δ that has a Δ*G*_hyd_: +1.2 kcal/mol and makes a direct hydrogen bond with Asn140, which is also expected to drive affinity. However, for the basic piperidine to make a direct salt bridge interaction with Asp144 and achieve BD1 domain selectivity, the energetically favorable water site α must be displaced. It is hypothesized that the energy penalty in displacing this highly stabilized water molecule offsets any benefit of the salt bridge interaction with Asp144 and explains the comparable BRD4 BD1 potency between (*S*)-**12** and **31**. This also sheds some light on the decreased BRD4 BD1 potency of molecules such as **32**, **33**, **35** and **36**, which will occupy the same water sites as **31** without making a beneficial interaction with Asp144 to offset the energy penalty of occupying water site α.

### Profile of **31**

With **31** meeting the predetermined BRD4 BD1/BD2 potency and selectivity targets, the compound progressed into downstream profiling assays to gain a more complete picture of both the BET bromodomain and non-BET bromodomain selectivity (Table 3).

**Table 3:**
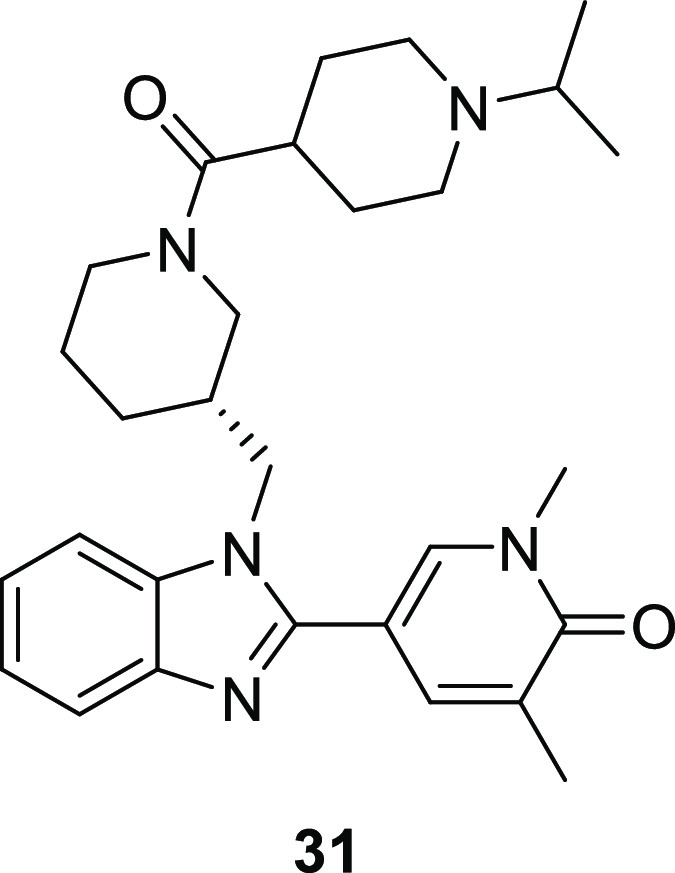
Broader Bromodomain Profile of **31** in Two Assay Formats

	TR-FRET pIC_50_		fold selectivity	BROMOscan p*K*_d_		fold selectivity
bromodomain	BD1	BD2		BD1	BD2	
BRD2	8.0	5.4	400	8.5	5.5	1000
BRD3	7.7	5.1	400	8.4	5.8	400
BRD4	7.8	4.8	1000	8.1	5.6	316
BRDT	7.3	4.5	630	8.1	5.5	400
EP300					5.0	1200
20 non-BET bromodomains					≤4.8	≥2000

The BRD4 BD1/BD2 TR-FRET assay had been used routinely for this optimization work, and in order to better understand the BET BD1 domain selectivity profile, **31** was tested in TR-FRET BRD2, BRD3, and BRDT assays. Similar levels of BD1 and BD2 potency were observed across the four isoforms, with at least 400-fold BD1 domain selectivity over BD2. The selectivity was also determined in the BROMOscan assay, which showed comparable potencies and BET BD1 domain selectivity across the assay formats.^52^ The broader non-BET bromodomain selectivity was also determined in the BROMOscan assay, with the closest off-target being EP300 with 1200-fold selectivity compared to BRD4 BD1 p*K*_d_: 8.1. Compound **31** also demonstrated ≥2000-fold selectivity over all 20 non-BET bromodomains tested (Table S2). Screening **31** in the GSK-enhanced cross-screening panel of pharmacologically relevant off-targets did not show any activity at the concentrations tested (pXC_50_ < 4.6, XC_50_ > 25 μM) (Table S4). Despite high levels of cellular activity, **31** had a low passive permeability of <3 nm/s as measured by an artificial membrane permeability assay, indicative of a risk of poor oral bioavailability.

Based on the high BET BD1 affinity, selectivity over not just BET BD2, but also the wider human bromodomain family and off-target liabilities, demonstration of cellular target engagement*via* inhibition of MCP-1 secretion in the LPS-stimulated hWB assay, PFI: 4.7, and high aqueous solubility, **31** (GSK023) was chosen as a BET BD1 domain-selective *in vitro* chemical probe. This molecule sits along with other reported structurally distinct high-quality BET BD1 domain-selective chemical probes, such as GSK789, for use in biological studies.^12^

**Figure 8:**
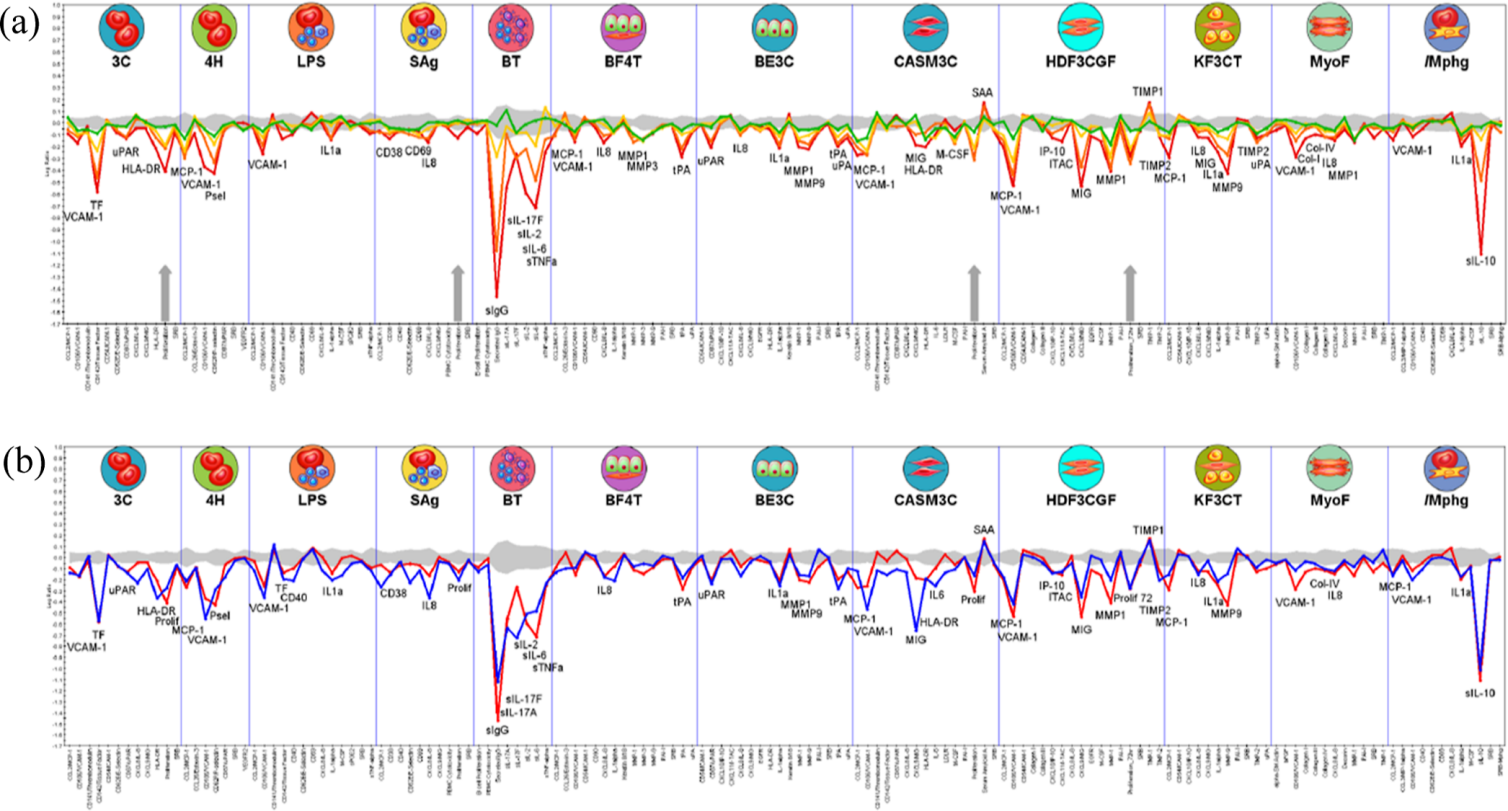
(a) BioMAP profile of **31** [10 μM (red), 2.5 μM (orange), 630 nM (yellow), and 160 nM (green)] in the Diversity PLUS Panel. The *X*-axis lists the quantitative protein-based biomarker readouts measured in each system. The *Y*-axis represents a log-transformed ratio of the biomarker readouts for the drug-treated sample (*n* = 1) over vehicle controls (*n* ≥ 6). The gray region around the *Y*-axis represents the 95% significance envelope generated from historical vehicle controls. Biomarker activities are annotated when two or more consecutive concentrations change in the same direction relative to vehicle controls, are outside of the significance envelope, and have at least one concentration with an effect size >20% (log_10_ ratio >0.1). Biomarker key activities are described as modulated if these activities increase in some systems but decrease in others. No cytotoxicity was observed at the concentrations tested, and antiproliferative effects are indicated by a thick gray arrow above the *X*-axis; (b) As (a), but with **31** (10 μM, red) and (+)-JQ1 (120 nM, blue).

The phenotypic profile of **31** was further investigated through the BioMAP Diversity PLUS platform, which contains 12 multiplex human primary-cell-based assays that model various human disease states.^53^ The 148 biomarker readouts include cell surface receptors, cytokines, chemokines, matrix molecules, and enzymes, which enable a comparison of holistic profiles to known mechanisms of action.^54^ Testing **31** across a range of concentrations from 10 μM to 160 nM revealed antiproliferative effects in human primary T cells, coronary artery smooth muscle cells, endothelial cells, and fibroblasts (Figure 8a). A variety of immune-modulatory biomarkers were impacted, including decreases in the levels of MCP-1, IL-8, IL-1, IL-10, M-CSF, and IL-6. An unsupervised search for mathematically similar compound profiles from the BioMAP Reference Database revealed that **31** is most similar to pan-BET inhibitor (+)-JQ1 with a Pearson’s correlation coefficient of 0.853 (Figure 8b).^7^

## Conclusions

In conclusion, we report a structure-guided approach to identify a structurally differentiated, high-quality BET BD1 chemical probe. A lipophilic efficiency analysis was used to uncover an efficient and BD1-biased benzimidazole substructure from the GSK collection of BRD4 BD1/BD2 screening data. X-ray crystallography analysis was used to guide the medicinal chemistry design strategy to target a conserved Asp/His switch to drive BD1 domain selectivity *via* a salt-bridge interaction, with metadynamics simulation and WaterMap analysis used to understand the observed SAR. These efforts resulted in chemical probe **31** bearing a basic piperidine, which makes a direct salt bridge with Asp144 and clashes with His433, driving high levels of BD1 domain selectivity (300–1000-fold depending on protein and assay format) across the BET bromodomain family. Further *in vitro* profiling demonstrated cellular target engagement, ≥1200-fold selectivity over all non-BET bromodomains and broader liability targets tested, and a phenotypic fingerprint indicative of BET bromodomain inhibition.

## Experimental Section

### Physicochemical Properties

ChromatographicLog*D* at pH 7.4, artificial membrane permeability, CLND solubility, and CAD solubility were measured using published protocols.^55^

### Chemistry Methods

All solvents were purchased from Sigma-Aldrich (anhydrous solvents), and commercially available reagents were used as received. All reactions were followed by TLC analysis (TLC plates GF254, Merck) or LCMS (liquid chromatography mass spectrometry) using a Waters ZQ instrument. NMR spectra were recorded at ambient temperature unless otherwise stated by using standard pulse methods on the following spectrometers and signal frequencies: Bruker AV-400 (^1^H = 400 MHz, ^13^C = 100.6 MHz). Chemical shifts are reported in parts per million and are referenced to tetramethylsilane (TMS) or the following solvent peaks: CDCl_3_ (^1^H = 7.27 ppm) and DMSO-*d*_6_ (^1^H = 2.50 ppm, ^13^C = 39.51 ppm). Coupling constants are quoted to the nearest 0.1 Hz, and multiplicities are given by the following abbreviations and combinations thereof: s (singlet), d (doublet), t (triplet), q (quartet), m (multiplet), and br (broad). Column chromatography was performed on prepacked silica gel columns using biotage SP4, Isolera One, or Teledyne ISCO apparatus. High-resolution mass spectra (HRMS) were recorded on a Micromass Q-Tof Ultima hybrid quadrupole time-of-flight mass spectrometer, with analytes separated on an Agilent 1100 Liquid Chromatograph equipped with a Phenomenex Luna C18(2) reversed phase column (100 × 2.1 mm, 3 μm packing diameter). LC conditions were 0.5 mL/min flow rate, 35 °C, injection volume 2–5 μL. Gradient elution with (A) H_2_O containing 0.1% (v/v) formic acid and (B) acetonitrile containing 0.1% (v/v) formic acid. Gradient conditions were initially 5% B, increasing linearly to 100% B over 6 min, remaining at 100% B for 2.5 min and then decreasing linearly to 5% B over 1 min, followed by an equilibration period of 2.5 min prior to the next injection. LCMS analysis was carried out on a Waters Acquity UPLC instrument equipped with a BEH column (50 mm × 2.1 mm, 1.7 μm packing diameter) and Waters micromass ZQ MS using alternate-scan positive and negative electrospray. Analytes were detected at a summed UV wavelength of 210–350 nm. The liquid-phase methods used were:

Formic–40 °C, 1 mL/min flow rate. Gradient elution with the mobile phases consisted of (A) H_2_O containing 0.1% volume/volume (v/v) formic acid and (B) acetonitrile containing 0.1% (v/v) formic acid. Gradient conditions were initially 1% B, increasing linearly to 97% B over 1.5 min, remaining at 97% B for 0.4 min, then increasing to 100% B over 0.1 min.

Formic B–35 °C, 0.6 mL/min flow rate. Gradient elution with the mobile phases as (A) H_2_O containing 0.1% volume/volume (v/v) formic acid and (B) acetonitrile containing 0.1% (v/v) formic acid. Gradient conditions were initially 3% B, increasing to 98% between 0.4 and 3.2 min, then dropping to 3% B between 3.8 and 4.2 min and maintaining at 3% B until 4.5 min.

High pH–40 °C, 1 mL/min flow rate. Gradient elution with the mobile phases was as follows: (A) 10 mM aqueous ammonium bicarbonate solution, adjusted to pH 10 with 0.88 M aqueous ammonia and (B) acetonitrile. Gradient conditions were initially 1% B, increasing linearly to 97% B over 1.5 min, remaining at 97% B for 0.4 min, then increasing to 100% B over 0.1 min.

Preparative HPLC was carried out with a Combiflash EZ prep HPLC machine and was conducted on an Xselect CSH C18 reverse phase column (100 mm × 30 mm i.d., 5 μm packing diameter) at ambient temperature. The solvents employed were 10 mM ammonium bicarbonate, adjusted to pH 10 with ammonia in water (solvent A) and acetonitrile (solvent B). The CombiFlash Rf uses RFID (Radio Frequency Identification) technology to automate the setting of the parameters for purification runs and fraction collection. The system is equipped with a UV variable dual–wavelength and a Foxy fraction collector, enabling automated peak cutting, collection, and tracking.

Mass-directed automatic purification (MDAP) was carried out using a Waters ZQ MS using alternate-scan positive and negative electrospray and a summed UV wavelength of 210–350 nm. Two liquid-phase methods were used:

The Formic–Sunfire C18 column (100 × 19 mm, 5 μm packing diameter, 20 mL/min flow rate) or the Sunfire C18 column (150 × 30 mm, 5 μm packing diameter, 40 mL/min flow rate). Gradient elution at ambient temperature with the mobile phases as (A) H_2_O containing 0.1% volume/volume (v/v) formic acid and (B) acetonitrile containing 0.1% (v/v) formic acid.

The high pH–Xbridge C18 column (100 mm × 19 mm, 5 μm packing diameter, 20 mL/min flow rate) or the Xbridge C18 column (150 mm × 30 mm, 5 μm packing diameter, 40 mL/min flow rate). Gradient elution at ambient temperature with the mobile phases as (A) 10 mM aqueous ammonium bicarbonate solution, adjusted to pH 10 with 0.88 M aq ammonia and (B) acetonitrile.

The purity of all compounds tested was determined by LCMS and ^1^H NMR to be >95%.

#### (*R*)-1-(3-(((2-Nitrophenyl)amino)methyl)piperidin-1-yl)ethan-1-one ((*R*)-**11**))

1-Fluoro-2-nitrobenzene (0.11 mL, 1.04 mmol), DIPEA (0.6 mL, 3.44 mmol), and (*R*)-1-(3-(aminomethyl)piperidin-1-yl)ethanone (342 mg, 2.19 mmol) were suspended in NMP (1 mL). The vial was sealed and heated in a Biotage Initiator microwave for 1 h at 200 °C using a very high absorption setting. The mixture was allowed to reach rt and then diluted with H_2_O (40 mL) and extracted with EtOAc (5 × 40 mL). The organic layers were combined and dried over a hydrophobic frit, and the solvent was removed under reduced pressure. The resultant residue was taken up into CH_2_Cl_2_ (5 mL), purified by column chromatography on a 40 g silica column, and eluted with 0–100% cyclohexane/EtOAc. The desired fractions were combined, and the solvent was removed under reduced pressure to give a residue, which was dried in a vacuum oven overnight to give (*R*)-**11** as a crude orange gum (493 mg), which was used without further purification. LCMS (high pH) (M + H)^+^ = 278.1, *R*_t_ = 0.98 min (96%).

#### (*S*)-1-(3-(((2-Nitrophenyl)amino)methyl)piperidin-1-yl)ethan-1-one ((*S*)-**11**))

(*S*)-1-(3-(Aminomethyl)piperidin-1-yl)ethanone (342 mg, 2.19 mmol), DIPEA (0.60 mL, 3.44 mmol), and 1-fluoro-2-nitrobenzene (0.11 mL, 1.04 mmol) were suspended in NMP (1 mL). The vial was sealed and then heated in a Biotage Initiator microwave for 1 h at 200 °C using a very high absorption setting. The mixture was allowed to reach rt and then diluted with H_2_O (40 mL) and extracted with EtOAc (5 × 40 mL). The organic layers were combined, dried over a hydrophobic frit, and the solvent was removed under reduced pressure. The residue was taken up into DCM (5 mL) and purified by column chromatography on a 40 g silica column and eluted with 0–100% cyclohexane/EtOAc. The desired fractions were combined, and the solvent was removed under reduced pressure to give (*S*)-**11** as a crude orange oil, which was used directly without further purification (323 mg). LCMS (high pH) (M + H)^+^ = 278.1, *R*_t_ = 0.98 min (88%).

#### (*R*)-5-(1-((1-Acetylpiperidin-3-yl)methyl)-1*H*-benzo[*d*]imidazole-2-yl)-1,3-dimethylpyridin-2(1*H*)-one ((*R*)-**12**)

(*R*)-**11** (250 mg, 0.90 mmol), Na_2_S_2_O_4_ (314 mg, 1.80 mmol), and 1,5-dimethyl-6-oxo-1,6- dihydropyridine-3-carbaldehyde (191 mg, 1.26 mmol) were suspended in EtOH (1.5 mL) and H_2_O (0.75 mL). The vial was sealed and then heated in a Biotage Initiator microwave oven for 65 min at 100 °C using a very high absorption setting. The reaction mixture was cooled to rt. Further, Na_2_S_2_O_4_ (314 mg, 1.80 mmol) was added, and the reaction mixture was heated in a Biotage Initiator microwave for 65 min at 100 °C using a very high absorption setting. Following cooling to rt, the reaction mixture was diluted with sat. NaHCO_3_ (30 mL). The separated aqueous phase was extracted with CH_2_Cl_2_ (4 × 30 mL), and the combined organic layers were dried over a hydrophobic frit. The solvent was removed under reduced pressure, and the residue was taken up into CH_2_Cl_2_ (5 mL) and then purified by column chromatography on a 40 g silica cartridge and eluted with 0–100% cyclohexane/EtOAc. The desired fractions were combined, and the solvent was removed under reduced pressure to give (*R*)-**12** as a white solid (113 mg, 33%). ^1^H NMR (400 MHz, 393 K, DMSO-*d*_6_) δ 7.97 (1H br s), 7.53–7.69 (3H, m), 7.18–7.31 (2H, m), 4.19–4.33 (2H, m), 3.64–3.84 (2H, m), 3.60 (3H, s), 2.84–2.95 (1H, m), 2.15 (3H, s), 1.93–2.07 (1H, m), 1.85 (3H, s), 1.47–1.69 (2H, m), 1.14–1.37 (3H, m); LCMS (high pH) (M + H)^+^ = 379.2, *R*_t_ = 0.77 min (100%).

#### (*S*)-5-(1-((1-Acetylpiperidin-3-yl)methyl)-1*H*-benzo[*d*]imidazole-2-yl)-1,3-dimethylpyridin-2(1*H*)-one ((*S*)-**12**)

(*S*)-**11** (150 mg, 0.54 mmol), Na_2_S_2_O_4_ (113 mg, 0.65 mmol), and 1,5-dimethyl-6-oxo-1,6- dihydropyridine-3-carbaldehyde (114 mg, 0.76 mmol) were suspended in EtOH (1.5 mL) and H_2_O (0.75 mL). The vial was sealed and then heated in a Biotage Initiator microwave for 65 min at 100 °C using a very high absorption setting. The reaction mixture was allowed to cool to rt, and further Na_2_S_2_O_4_ (113 mg, 0.65 mmol) was added; the reaction mixture was heated in a Biotage Initiator microwave for 65 min at 100 °C using a very high absorption setting. The reaction mixture was allowed to cool to rt and then diluted with sat. aq NaHCO_3_ (30 mL). The separated aqueous phase was extracted with CH_2_Cl_2_ (4 × 30 mL), and the combined organic layers were dried over a hydrophobic frit. The solvent was removed under reduced pressure, and the resultant residue was taken up into CH_2_Cl_2_ (5 mL) and purified by column chromatography on a 40 g silica cartridge and eluted with 0–100% cyclohexane/EtOAc. The desired fractions were combined, and the solvent was removed under reduced pressure. The resultant residue was dissolved in 1:1 MeOH/DMSO (0.6 mL) and purified by MDAP (high pH). The solvent was removed under reduced pressure to give (*S*)-**12** (44 mg, 22%). Analytical data are as for (*R*)-**12** above.

#### (*S*)-5-(1-((1-Acetylpiperidin-3-yl)methyl)-5-bromo-1*H*-benzo[*d*]imidazole-2-yl)-1,3-dimethylpyridin-2(1*H*)-one (**14**)

To a solution of **20** (3.50 g, 7.75 mmol) in THF (100 mL) was added NEt_3_ (1.08 mL, 7.75 mmol) followed by AcCl (0.55 mL, 7.75 mmol) at 0 °C. The mixture was stirred at rt for 1 h prior to being diluted with H_2_O (100 mL) and EtOAc (100 mL). The separated organic phase was washed with H_2_O (3 × 100 mL); the combined organic phase was dried over Na_2_SO_4_ and then evaporated under vacuum to give a yellow solid. The solid was washed with Et_2_O and then dried under a vacuum to give **14** as a yellow solid (1.80 g, 50%). ^1^H NMR (400 MHz, 363 K, DMSO-*d*_6_) δ 8.05 (1H, s), 7.78 (1H, d, *J* = 1.8 Hz), 7.64–7.70 (1H, m), 7.56–7.64 (1H, m), 7.37 (1H, dd, *J* = 8.6, 2.0 Hz), 4.14–4.34 (2H, m), 3.56 (3H, s), 2.96–3.02 (4H, m), 2.11 (3H, s), 1.73–1.99 (4H, m), 1.43–1.57 (2H, m), 1.05–1.29 (2H, m); LCMS (formic method B) (M + H)^+^ = 457.1, 459.1, *R*_t_ = 1.74 min (99%).

#### *tert*-Butyl (*S*)-3-(((2-Nitrophenyl)amino)methyl)piperidine-1-carboxylate (**15**)

K_2_CO_3_ (484 mg, 3.50 mmol) and 1-fluoro-2-nitrobenzene (246 μL, 2.33 mmol) were added to a stirring solution of *tert*-butyl (*S*)-3-(aminomethyl)piperidine-1-carboxylate (500 mg, 2.33 mmol) in DMF (10 mL). The reaction was stirred at 100 °C under N_2_ for 4 h. After being cooled to rt, the reaction mixture was concentrated *in vacuo*. The residue was diluted with and partitioned between EtOAc (20 mL) and H_2_O (20 mL), and the phases were separated. The aqueous phase was further extracted with EtOAc (20 mL). The combined organic phases were washed with a 5% (by weight) aqueous solution of LiCl (30 mL), dried by passing through a hydrophobic frit, and concentrated *in vacuo*. The resultant material was then purified by chromatography, eluting with 0–20% EtOAc in cyclohexane. Fractions containing the desired product were concentrated *in vacuo* to give **15** as an orange gum (763 mg, 98%). ^1^H NMR (400 MHz, CDCl_3_) δ 8.18 (1H, dd, *J* = 8.6, 1.7 Hz), 8.09–8.16 (1H, m), 7.41–7.47 (1H, m), 6.84 (1H, dd, *J* = 8.8, 1.0 Hz), 6.63–6.69 (1H, m), 3.99 (1H, br s), 3.85 (1H, dt, *J* = 13.1, 4.0 Hz), 3.23–3.32 (1H, m), 3.14–3.22 (1H, m), 2.91–3.02 (1H, m), 2.80 (1H, br s), 1.87–2.00 (2H, m), 1.65–1.75 (1H, m), 1.44–1.56 (10H, m), 1.28–1.40 (1H, m); LCMS (high pH) (M + H)^+^ = 336.3, *R*_t_ = 1.36 min (93%).

#### *tert*-Butyl (*S*)-3-(((4-Bromo-2-nitrophenyl)amino)methyl)piperidine-1-carboxylate (**16**)

A mixture of 4-bromo-1-fluoro-2-nitrobenzene (2.00 g, 9.09 mmol), (*S*)-*tert*-butyl 3- (aminomethyl)piperidine-1-carboxylate (1.95 g, 9.09 mmol), and K_2_CO_3_ (2.51 g, 18.18 mmol) was heated in DMF (10 mL) at 80 °C for 3 h. Upon cooling to rt, the reaction mixture evaporated under reduced pressure and was then diluted with H_2_O (50 mL) and EtOAc (50 mL). The separated aqueous phase was extracted with EtOAc (50 mL), and the combined organic phase was passed through a hydrophobic frit and then evaporated under reduced pressure to give **16** as a crude orange solid (4.34 g), which was used without further purification. LCMS (high pH) (M)^−^ = 412.2, 414.2, *R*_t_ = 1.48 min (98%).

#### (*S*)-3-((2-(1,5-Dimethyl-6-oxo-1,6-dihydropyridin-3-yl)-*H*-benzo[*d*]imidazole-1-yl)methyl)piperidine-1-carboxylate (**17**)

**15** (373 mg, 1.11 mmol), 1,5-dimethyl-6-oxo-1,6-dihydropyridine-3-carbaldehyde (202 mg, 1.34 mmol), EtOH (7 mL), and H_2_O (3.5 mL) were combined. The reaction mixture was heated to 80 °C, after which sodium dithionite (581 mg, 3.34 mmol) was added. The reaction mixture was stirred at 100 °C under N_2_ for 1 h. After being cooled to rt, the reaction mixture was diluted with and partitioned between EtOAc (20 mL) and H_2_O (20 mL), and the phases were separated. The aqueous phase was further extracted with EtOAc (20 mL). The combined organic phases were washed with brine (20 mL), dried by being passed through a hydrophobic frit, and concentrated *in vacuo*. This resultant residue was dried under a high vacuum for 16 h to give **17** as a yellow gum (383 mg, 79%). ^1^H NMR (400 MHz, CDCl_3_) δ 7.73–7.78 (2H, m), 7.49–7.51 (1H, m), 7.35–7.40 (1H, m), 7.29–7.34 (2H, m), 4.06–4.20 (2H, m), 3.77–3.87 (1H, m), 3.65 (3H, s), 2.77 (1H,, br. t *J* = 12.2 Hz), 2.53 (1H, dd, *J* = 13.0, 10.0 Hz), 2.25 (3H, s), 1.99–2.11 (1H, m), 1.56 (2H, apparent br. d, *J* = 10.8 Hz), 1.23–1.48 (11H, m), 0.99–1.12 (1H, m); LCMS (high pH) (M + H)^+^ = 437.4, *R*_t_ = 1.07 min (98%).

#### *tert*-Butyl (*S*)-3-((5-Bromo-2-(1,5-dimethyl-6-oxo-1,6-dihydropyridin-3-yl)-1*H*-benzo[*d*]imidazole-1-yl)methyl)piperidine-1-carboxylate (**18**)

A mixture of **16** (10.00 g, 24.14 mmol), 1,5-dimethyl-6-oxo-1,6-dihydropyridine-3-carbaldehyde (3.65 g, 24.14 mmol), and Na_2_S_2_O_4_ (7.54 g, 43.3 mmol) was heated in EtOH (100 mL) and H_2_O (20 mL) at 100 °C for 16 h. Upon cooling to rt, the reaction mixture was diluted with H_2_O (100 mL) and EtOAc (100 mL). The separated organic phase was washed with H_2_O (3 × 50 mL) and dried over Na_2_SO_4_ to give crude 15 as a pale yellow solid (5.00 g), which was used without further purification. LCMS (formic method B) (M + H)^+^ = 515.3, 517.3, and *R*_t_ = 2.29 min (83%).

#### (*R*)-1,3-Dimethyl-5-(1-(piperidin-3-ylmethyl)-1*H*-benzo[*d*]imidazole-2-yl)pyridin-2(1*H*)-one (**19**)

**17** (365 mg, 0.84 mmol) was dissolved in a solution of HCl in 1,4–dioxane (4 M, 4 mL, 16 mmol) and MeOH (1 mL). The reaction mixture was stirred at rt in air for 1 h and then diluted with MeOH (20 mL). The solution was added to an SCX 20 g column (pre–wet with MeOH) and allowed to settle by gravity. The SCX column was then washed with MeOH (80 mL) and then 2 M NH_3_ in MeOH (80 mL) under vacuum. The basic filtrate was concentrated under reduced pressure to give **19** as a yellow gum (283 mg, 96%). ^1^H NMR (400 MHz, CDCl_3_) δ 7.80 (1H, d, *J* = 2.4 Hz), 7.73–7.77 (1H, m), 7.53–7.55 (1H, m), 7.36–7.41 (1H, m), 7.26–7.31 (2H, integrates as 3H due to CHCl_3_ peak obscuring signal, m), 4.17 (1H, dd, *J* = 14.7, 8.3 Hz), 4.09 (1H, dd, *J* = 14.2, 6.4 Hz), 3.64 (3H, s), 2.91 (1H, dt, *J* = 12.1, 4.0 Hz), 2.76 (1H, dd, *J* = 11.7, 3.4 Hz), 2.52–2.61 (1H, m), 2.31 (1H, dd, *J* = 11.7, 9.8 Hz), 2.24 (3H, s), 2.02–2.21 (3H, m), 1.57–1.66 (1H, integrates as 2H due to the overlapping water peak, m), 1.32–1.45 (1H, m), 1.06–1.18 (1H, m). LCMS (high pH) (M + H)^+^ = 337.3, *R*_t_ = 0.73 min (100%).

#### (*R*)-5-(5-Bromo-1-(piperidin-3-ylmethyl)-1*H*-benzo[*d*]imidazole-2-yl)-1,3-dimethylpyridin-2(1*H*)-one Hydrochloride Salt (**20**)

To a solution of **18** (5.00 g, 9.70 mmol) in MeOH (100 mL) was added HCl (68 mL, 4 M in 1,4-dioxane, 272 mmol) at 0 °C. Following stirring at rt for 2 h, the solvent was evaporated under reduced pressure, and the resultant residue was triturated with EtOAc. Evaporation under reduced pressure gave crude **18** as a gray solid (3.50 g), which was used without further purification. LCMS (formic method B) (M + H)^+^ = 415.2, 417.3, and *R*_t_ = 1.36 min (70%).

#### (*S*)-5-(1-((1-(4-Aminobutanoyl)piperidin-3-yl)methyl)-1*H*-benzo[*d*]imidazole-2-yl)-1,3-dimethylpyridin-2(1*H*)-one (**21**)

DIPEA (0.34 mL, 1.93 mmol) was added to a solution of HATU (184 mg, 0.48 mmol) and 4-((*tert*-butoxycarbonyl)amino)butanoic acid (79 mg, 0.39 mmol) in DMF (1.5 mL). The reaction mixture was stirred at rt for 15 min, after which a solution of **19** (130 mg, 0.39 mmol) in DMF (1.5 mL) was added. Following stirring at rt for 2 h, the reaction mixture was dissolved in EtOAc (20 mL) and 5% aq LiCl (20 mL). The separated aqueous phase was washed with EtOAc (20 mL), and the combined organic phase was washed with 5% aq LiCl (3 × 20 mL). The organic phase was passed through a hydrophobic frit and evaporated to give a brown gum (229 mg). The gum was dissolved in 4 M HCl in 1,4-dioxane (4 mL, 16.00 mmol), and the mixture was stirred for 1 h. MeOH (20 mL) was added, and the mixture was loaded onto a 20 g SCX column (prewetted with MeOH) and allowed to settle by gravity. The column was washed under a vacuum with MeOH (80 mL) and then 2 M NH_3_ in MeOH (80 mL). The collected methanol ammonia solution was evaporated under reduced pressure to give a white solid. The solid was dissolved in 1:1 MeOH/DMSO (1 mL) and purified by MDAP (high pH). The appropriate fractions were combined and evaporated under a stream of N_2_ to give **21** as a white gum (10 mg 6% over two steps). ^1^H NMR (400 MHz, CDCl_3_) δ 7.74–7.87 (2H, m), 7.50–7.54 (1H, m), 7.37–7.45 (1H, m), 7.29–7.35 (2H, m), 4.04–4.37 (3H, m), 3.64–3.70 (4H, m), 3.36–3.43 (1H, m), 2.98–3.06 (1H, m), 2.77 (1H, br. t, *J* = 6.8 Hz), 2.50–2.69 (2H, m), 2.36 (1H, br. t, *J* = 7.3 Hz), 2.26 (3H, s), 1.95–2.14 (2H, m), 1.74–1.85 (1H, m), 1.50–1.69 (3H, m), 1.00–1.39 (3H, m); LCMS (high pH) (M + H)^+^ = 422.4, *R*_t_ = 0.72 min (99%).

#### (*S*)-5-(1-((1-(Azetidine-3-carbonyl)piperidin-3-yl)methyl)-1*H*-benzo[*d*]imidazole-2-yl)-1,3-dimethylpyridin-2(1*H*)-one (**22**)

DIPEA (78 μL, 0.45 mmol) was added to a stirred solution of 1-(*tert*-butoxycarbonyl)azetidine-3-carboxylic acid (30 mg, 0.15 mmol) and HATU (71 mg, 0.19 mmol) in DMF (0.5 mL) at rt. Following stirring at rt for 10 min, a solution of **19** (50 mg, 0.15 mmol) in DMF (0.5 mL) was added. The reaction mixture was stirred at rt for 16 h, and then additional 1-(*tert*-butoxycarbonyl)azetidine-3-carboxylic acid (30 mg, 0.15 mmol) and HATU (71 mg, 0.19 mmol) were added, and the reaction mixture was stirred at rt for a further 4 h. The reaction mixture was diluted with CH_2_Cl_2_ (20 mL) and 10% aq LiCl (20 mL). The separated aqueous phase was extracted with CH_2_Cl_2_ (20 mL), and the combined organic phase was washed with 10% aq LiCl (2 × 20 mL), passed through a hydrophobic frit, and evaporated under reduced pressure to give a brown oil (128 mg). The oil was dissolved in 4 M HCl in 1,4-dioxane (3 mL) and MeOH (1 mL), and the mixture was stirred at rt for 4 h. The mixture was concentrated under reduced pressure; the resultant residue dissolved in 1:1 MeOH/DMSO (1 mL) and then purified by MDAP (high pH). The appropriate fractions were combined and extracted with CH_2_Cl_2_ (2 × 20 mL). The combined organic phase was passed through a hydrophobic frit and evaporated under reduced pressure to give compound **22** as a yellow solid (20 mg, 32% over two steps). ^1^H NMR (400 MHz, 393 K, DMSO-*d*_6_) δ 7.99 (1H, d, *J* = 2.4 Hz), 7.63–7.68 (2H, m), 7.55–7.59 (1H, m), 7.21–7.29 (2H, m), 4.19–4.32 (2H, m), 3.62–3.69 (1H, m), 3.53–3.61 (4H, m), 3.32–3.51 (3H, m), 2.70–2.93 (3H, integrates as 12H due to the overlapping water peak, m), 2.66 (1H, dd, *J* = 13.0, 10.0 Hz), 2.15 (3H, s), 1.87–1.98 (1H, m), 1.53–1.61 (2H, m), 1.13–1.31 (3H, m); LCMS (high pH) (M + H)^+^ = 420.4, *R*_t_ = 0.67 min (100%).

#### 5-(1-(((*S*)-1-((1*r*,3*S*)-3-Aminocyclobutane-1-carbonyl)piperidin-3-yl)methyl)-1*H*-benzo[*d*]imidazole-2-yl)-1,3-dimethylpyridin-2(1*H*)-one (**23**)

DIPEA (76 μL, 0.36 mmol) was added to a stirring solution of (1*r*,3*r*)-3-((*tert*-butoxycarbonyl)amino)cyclobutane-1-carboxylic acid (32 mg, 0.15 mmol) and HATU (71 mg, 0.19 mmol) in DMF (0.5 mL) at rt. Following stirring at rt for 5 min, a solution of **19** (50 mg, 0.15 mmol) in DMF (0.5 mL) was added. The reaction mixture was stirred at rt under air for 0.5 h, and then CH_2_Cl_2_ (20 mL) and 10% aq LiCl (20 mL) were added. The separated aqueous phase was extracted with CH_2_Cl_2_ (20 mL); the combined organic phase was washed with 10% aq LiCl (3 × 20 mL), passed through a hydrophobic frit, and evaporated under reduced pressure to give a brown oil. The oil was dissolved in 4 M HCl in 1,4-dioxane (1 mL) and stirred at rt for 16 h. Following evaporation under reduced pressure, the resultant residue was dissolved in 1:1 MeOH/DMSO (2 mL) and purified by preparative HPLC, eluting with 15–100% MeCN/10 mM aq ammonium bicarbonate. The appropriate fractions were combined and extracted with CH_2_Cl_2_ (3 × 20 mL). The combined organic phase was passed through a hydrophobic frit and evaporated under reduced pressure to give **23** as white solid (23 mg, 36% over two steps). ^1^H NMR (400 MHz, 393 K, DMSO-*d*_6_) δ 7.98 (1H, d, *J* = 2.4 Hz), 7.63–7.68 (2H, m), 7.55–7.58 (1H, m), 7.20–7.29 (2H, m), 4.18–4.31 (2H, m), 3.70–3.86 (1H, m), 3.56–3.63 (4H, m), 3.28–3.36 (1H, m), 2.93–3.05 (1H, m), 2.70–2.89 (1H, integrates as 5H due to the overlapping water peak, m), 2.65 (1H, dd, *J* = 13.1, 9.9 Hz), 2.28–2.35 (1H, m), 2.17–2.25 (1H, m), 2.14 (3H, s), 1.85–1.97 (1H, m), 1.52–1.75 (5H, m), 1.12–1.30 (3H, m); LCMS (high pH) (M + H)^+^ = 434.4, *R*_t_ = 0.75 min (97%).

#### 1,3-Dimethyl-5-(1-(((*S*)-1-((*S*)-pyrrolidine-3-carbonyl)piperidin-3-yl)methyl)-1*H*-benzo[*d*]imidazole-2-yl)pyridin-2(1*H*)-one (**24**)

DIPEA (94 μL, 0.36 mmol) was added to a stirring solution of (*S*)-1-(*tert*-butoxycarbonyl)pyrrolidine-3-carboxylic acid (40 mg, 0.18 mmol) and HATU (88 mg, 0.23 mmol) in DMF (0.5 mL) at rt. Following stirring at rt for 10 min, a solution of **19** (62 mg, 0.18 mmol) in DMF (0.5 mL) was added. The reaction mixture was then stirred at rt for 66 h and then diluted with CH_2_Cl_2_ (20 mL) and 10% aq LiCl (20 mL). The separated aqueous phase was extracted with CH_2_Cl_2_ (20 mL). The separated aqueous phase was extracted with CH_2_Cl_2_ (20 mL), the combined organic phase was washed with 10% aq LiCl (3 × 20 mL), passed through a hydrophobic frit, and evaporated under reduced pressure. The resultant residue was dissolved in 4 M HCl in 1,4-dioxane (1 mL) and stirred at rt for 24 h. Following evaporation under reduced pressure, the resultant residue was dissolved in 1:1 MeOH/DMSO (1 mL) and purified by preparative HPLC, eluting with 0–100% MeCN/10 mM aq ammonium bicarbonate. The appropriate fractions were combined and extracted with CH_2_Cl_2_ (2 × 30 mL). The combined organic phase was passed through a hydrophobic frit and evaporated under reduced pressure to give **24** as a pale yellow solid (22 mg, 28% over two steps). ^1^H NMR (400 MHz, 393 K, DMSO-*d*_6_) δ 7.99 (1H, d, *J* = 2.5 Hz), 7.62–7.68 (2H, m), 7.56–7.59 (1H, m), 7.20–7.29 (2H, m), 4.20–4.33 (2H, m), 3.91 (1H, br. d, *J* = 12.5 Hz), 3.71–3.80 (1H, m), 3.59 (3H, s), 2.66–2.92 (7H, integrates as 13H due to the overlapping water peak, m), 2.14 (3H, s), 1.87–1.99 (1H, m), 1.69–1.79 (2H, m), 1.54–1.62 (2H, m), 1.14–1.33 (3H, m); LCMS (high pH) (M + H)^+^ = 434.4, *R*_t_ = 0.77 min (100%).

#### 5-(1-(((*S*)-1-((1*S*,3*S*)-3-Aminocyclopentane-1-carbonyl)piperidin-3-yl)methyl)-1*H*-benzo[*d*]imidazole-2-yl)-1,3-dimethylpyridin-2(1*H*)-one (**25**)

DIPEA (76 μL, 0.36 mmol) was added to a stirring solution of (1*S*,3*S*)-3-((*tert*-butoxycarbonyl)amino)cyclopentane-1-carboxylic acid (34 mg, 0.15 mmol) and HATU (71 mg, 0.19 mmol) in DMF (0.5 mL) at rt. Following stirring at rt for 5 min, a solution of **19** (50 mg, 0.15 mmol) in DMF (0.5 mL) was added. The reaction mixture was stirred at rt for 1 h and then diluted with CH_2_Cl_2_ (20 mL) and 10% aq LiCl (20 mL). The separated aqueous phase was extracted with CH_2_Cl_2_ (20 mL). The separated aqueous phase was extracted with CH_2_Cl_2_ (20 mL), the combined organic phase was washed with 10% aq LiCl (3 × 20 mL), passed through a hydrophobic frit, and evaporated under reduced pressure to give a brown oil. The oil was dissolved in 4 M HCl in 1,4-dioxane (1 mL) and stirred at rt for 16 h. Following evaporation under reduced pressure, the resultant residue was dissolved in 1:1 MeOH/DMSO (2 mL) and purified by preparative HPLC, eluting with 15–100% MeCN/10 mM aq ammonium bicarbonate. The appropriate fractions were combined and extracted with CH_2_Cl_2_ (2 × 20 mL). The combined organic phase was passed through a hydrophobic frit and evaporated under reduced pressure to give **25** as a white solid (29 mg, 44% over two steps). ^1^H NMR (400 MHz, 393 K, DMSO-*d*_6_) δ 7.99 (1H, d, *J* = 2.4 Hz), 7.62–7.68 (2H, m), 7.56–7.59 (1H, m), 7.20–7.29 (2H, m), 4.20–4.32 (2H, m), 3.90 (1H, br. d, *J* = 12.2 Hz), 3.73 (1H, br. d, *J* = 12.2 Hz), 3.59 (3H, s), 3.26–3.33 (1H, m), 2.65–3.01 (3H, integrates as 7H due to the overlapping water peak, m), 2.14 (3H, s), 1.87–1.99 (1H, m), 1.54–1.82 (7H, m), 1.14–1.32 (5H, m). LCMS (high pH) (M + H)^+^ = 448.4, *R*_t_ = 0.78 min (99%).

#### (*S*)-5-(1-((1-(1*H*-Imidazole-5-carbonyl)piperidin-3-yl)methyl)-1*H*-benzo[*d*]imidazole -2-yl)-1,3-dimethylpyridin-2(1*H*)-one, Formic Acid Salt (**26**)

DIPEA (130 μL, 0.74 mmol) was added to a stirring solution of HATU (71 mg, 0.19 mmol) and 1*H*-imidazole-5-carboxylic acid (17 mg, 0.15 mmol) in DMF (0.5 mL) at rt. Following stirring at rt for 10 min, a solution of **19** (50 mg, 0.15 mmol) in DMF (0.5 mL) was added. The reaction mixture was stirred at rt under N_2_, additional 1*H*-imidazole-5-carboxylic acid (17 mg, 0.15 mmol) was added and stirring continued for a further 16 h under N_2_. CH_2_Cl_2_ (10 mL) and 5% aq LiCl (20 mL) were added. The separated aqueous phase was extracted with CH_2_Cl_2_ (2 × 10 mL). The separated aqueous phase was extracted with CH_2_Cl_2_ (20 mL), the combined organic phase was washed with 5% aq LiCl (10 mL), passed through a hydrophobic frit, and evaporated under reduced pressure. The resultant residue was dissolved in 1:1 MeOH/DMSO (1 mL) and purified by MDAP (high pH). The appropriate fractions were combined and extracted with CH_2_Cl_2_ (2 × 20 mL). The combined organic phase was passed through a hydrophobic frit and evaporated under reduced pressure. The resultant residue was dissolved in 1:1 MeOH/DMSO (1 mL) and purified by MDAP (formic). The appropriate fractions were combined and evaporated under reduced pressure to give **26** as a white solid (14 mg, 20%). ^1^H NMR (400 MHz, DMSO-*d*_6_) δ 8.22 (1H, br s), 7.91–7.98 (1H, m), 7.58–7.67 (2H, m), 7.14–7.58 (5H, m), 4.21–4.40 (4H, m), 3.53 (3H, s), 2.98–3.07 (1H, m), 2.80–2.91 (1H, m), 2.45–2.52 (1H, integrates as 10H due to the overlapping DMSO peak, m), 2.07–2.15 (3H, m), 1.98–2.07 (1H, m), 1.54–1.67 (2H, m), 1.18–1.39 (2H, m); LCMS (high pH) (M + H)^+^ = 431.4, *R*_t_ = 0.72 min (100%).

#### (*S*)-1,3-Dimethyl-5-(1-((1-(piperidine-4-carbonyl)piperidin-3-yl)methyl)-1*H*-benzo[*d*]imidazole-2-yl)pyridin-2(1*H*)-one (**27**)

DIPEA (130 μL, 0.74 mmol) was added to a solution of 1-(*tert*-butoxycarbonyl)piperidine-4-carboxylic acid (34 mg, 0.15 mmol) and HATU (71 mg, 0.19 mmol) in DMF (0.5 mL) at rt. Following stirring at rt for 10 min, a solution of 19 (50 mg, 0.15 mmol) in DMF (0.5 mL) was added and then stirred at rt for 1 h. The reaction mixture was diluted with EtOAc (20 mL) and 5% aq LiCl (20 mL). The separated aqueous phase was extracted with EtOAc (20 mL). The combined organic phase was washed with 5% aq LiCl (20 mL), passed through a hydrophobic frit, and evaporated under reduced pressure to give a brown oil. The oil was dissolved in 4 M HCl in 1,4-dioxane (0.5 mL). Following stirring at rt for 3 h under N_2_, the reaction was evaporated under reduced pressure. The resultant residue was dissolved in 1:1 MeOH/DMSO (1 mL) and purified by MDAP (high pH). The appropriate fractions were combined and extracted with CH_2_Cl_2_ (3 × 50 mL). The combined organic phase was passed through a hydrophobic frit and evaporated under reduced pressure to give 27 as a white solid (17 mg, 26% over two steps). ^1^H NMR (400 MHz, 393 K, DMSO-*d*_6_) δ 7.89–8.06 (1H, m), 7.61–7.68 (2H, m), 7.52–7.61 (1H, m), 7.17–7.31 (2H, m), 4.20–4.33 (2H, m), 3.83–3.99 (1H, m), 3.61–3.72 (1H, m), 3.59 (3H, s), 2.74–2.94 (4H, integrates as 7H due to the overlapping water peak, m), 2.64–2.73 (1H, m), 2.30–2.47 (3H, m), 2.14 (3H, s), 1.87–1.99 (1H, m), 1.54–1.64 (2H, m), 1.14–1.48 (6H, m). LCMS (high pH) (M + H)^+^ = 448.5, *R*_t_ = 0.80 min (100%).

#### 5-(1-(((*S*)-1-((1*r*,4*R*)-4-Aminocyclohexane-1-carbonyl)piperidin-3-yl)methyl)-1*H*-benzo [*d*]imidazole-2-yl)-1,3-dimethylpyridin-2(1*H*)-one (**28**)

DIPEA (76 μL, 0.36 mmol) was added to a stirred solution of (1*r*,4*s*)-4-((*tert*-butoxycarbonyl)amino)cyclohexane-1-carboxylic acid (36 mg, 0.15 mmol) and HATU (71 mg, 0.19 mmol) in DMF (0.5 mL) at rt. Following stirring at rt for 10 min, a solution of **19** (50 mg, 0.15 mmol) in DMF (0.5 mL). The reaction mixture was stirred at rt for 2 h and then diluted with CH_2_Cl_2_ (20 mL) and 10% aq LiCl (20 mL). The separated aqueous phase was extracted with CH_2_Cl_2_ (20 mL). The separated aqueous phase was extracted with CH_2_Cl_2_ (20 mL), the combined organic phase was washed with 10% aq LiCl (2 × 20 mL), passed through a hydrophobic frit, and evaporated under reduced pressure. The resultant residue was dissolved in 4 M HCl in 1,4-dioxane (1 mL) and stirred at rt for 16 h. Following evaporation under reduced pressure, the resultant residue was dissolved in 1:1 MeOH/DMSO (1 mL) and purified by preparative HPLC, eluting with 15–100% MeCN/10 mM aq ammonium bicarbonate. The appropriate fractions were combined and extracted with CH_2_Cl_2_ (2 × 20 mL). The combined organic phase was passed through a hydrophobic frit and evaporated under reduced pressure to give **28** as a white solid (11 mg, 16% over two steps). ^1^H NMR (400 MHz, 393 K, DMSO-*d*_6_) δ 7.98 (1H, d, *J* = 2.0 Hz), 7.62–7.67 (2H, m), 7.57 (1H, d, *J* = 7.3 Hz), 7.20–7.29 (2H, m), 4.20–4.32 (2H, m), 3.90 (1H, br. d, *J* = 12.7 Hz), 3.62–3.70 (1H, m), 3.59 (3H, s), 2.65–2.95 (2H, integrates as 9H due to the overlapping water peak, m), 2.25–2.34 (1H, m), 2.14 (3H, s), 1.88–1.99 (1H, m), 1.54–1.76 (5H, m), 1.33–1.53 (5H, m), 1.13–1.32 (5H, m); LCMS (high pH) (M + H)^+^ = 462.4, *R*_t_ = 0.82 min (98%).

#### 1,3-Dimethyl-5-(1-(((3*S*)-1-(1-methylpiperidine-3-carbonyl)piperidin-3-yl)methyl)-1*H*-benzo[*d*]imidazole-2-yl)pyridin-2(1*H*)-one (**29**)

DIPEA (130 μL, 0.74 mmol) was added to a solution of 1-methylpiperidine-3-carboxylic acid (21 mg, 0.15 mmol) and HATU (71 mg, 0.19 mmol) in DMF (0.5 mL) at rt. Following stirring at rt for 10 min, a solution of **19** (50 mg, 0.15 mmol) in DMF (0.5 mL) was added. The reaction mixture was stirred at rt for 1 h, and then EtOAc (20 mL) and 5% aq LiCl (20 mL) were added. The separated aqueous phase was extracted with EtOAc (20 mL), and the combined organic phase was washed with a 5% aq LiCl (20 mL), passed through a hydrophobic frit, and evaporated under reduced pressure. The combined aqueous phase was further extracted with CH_2_Cl_2_ (2 × 20 mL), the combined organic phase was passed through a hydrophobic frit, added to the evaporated residue from the EtOAc extraction, and concentrated under reduced pressure. The resultant residue was dissolved in 1:1 DMSO/MeOH (1 mL) and purified by MDAP (high pH). The appropriate fractions were combined and extracted with CH_2_Cl_2_ (2 × 30 mL). The combined organic phase was passed through a hydrophobic frit and evaporated under reduced pressure to give **29** as a white solid (29 mg, 42%). ^1^H NMR (400 MHz, 393 K, DMSO-*d*_6_) δ 7.94–7.97 (1H, m), 7.58–7.65 (2H, m), 7.55 (1H, d, *J* = 7.8 Hz), 7.17–7.26 (2H, m), 4.16–4.32 (2H, m), 3.80–3.90 (1H, m), 3.62–3.73 (1H, m), 3.56 (3H, s), 2.76–2.80 (1H, integrates as 13H due to the overlapping water peak, m), 2.62–2.70 (2H, m), 2.44–2.58 (2H, integrates as 24H due to the overlapping DMSO peak, m), 2.07–2.14 (6H, m), 1.72–1.96 (3H, m), 1.11–1.60 (8H, m); LCMS (high pH) (M + H)^+^ = 462.4, *R*_t_ = 0.83 min (100%).

#### (*S*)-1,3-Dimethyl-5-(1-((1-(1-methylpiperidine-4-carbonyl)piperidin-3-yl)methyl)-1*H*-benzo[*d*]imidazole-2-yl)pyridin-2(1*H*)-one (**30**)

DIPEA (78 μL, 0.45 mmol) was added to a stirred solution of 1-methylpiperidine-4-carboxylic acid (21 mg, 0.15 mmol) and HATU (71 mg, 0.19 mmol) in DMF (0.5 mL) at rt. Following stirring at rt for 10 min, a solution of **19** (50 mg, 0.15 mmol) in DMF (0.5 mL) was added. The reaction mixture was stirred at rt for 1 h, and then EtOAc (20 mL) and 5% aq LiCl (20 mL) were added. The separated aqueous phase was extracted with 4:1 CHCl_3_/IPA (3 × 20 mL). Then, the aqueous phase was salted by the addition of solid NaCl (approximately 2 g), and the aqueous phase was further extracted with 4:1 CHCl_3_/IPA (3 × 20 mL). The combined organic phases were washed with brine (2 × 20 mL), passed through a hydrophobic frit, and evaporated under reduced pressure. The resultant residue was dissolved in 1:1 MeOH/DMSO (1 mL) and purified by MDAP (high pH). The appropriate fractions were combined and extracted with CH_2_Cl_2_ (2 × 40 mL). The combined organic phase was passed through a hydrophobic frit and evaporated under reduced pressure to give **30** as a yellow solid (19 mg, 28%). ^1^H NMR (400 MHz, 393 K, DMSO-*d*_6_) δ 7.98 (1H, d, *J* = 2.2 Hz), 7.62–7.67 (2H, m), 7.55–7.60 (1H, m), 7.20–7.29 (2H, m), 4.20–4.32 (2H, m), 3.90 (1H, br. d, *J* = 12.7 Hz), 3.65 (1H, br. d, *J* = 12.7 Hz), 3.59 (3H, s), 2.75–2.86 (1H, integrates as 6H due to the overlapping water peak, m), 2.62–2.73 (3H, m), 2.11–2.24 (7H, m), 1.87–1.99 (1H, m), 1.72–1.86 (2H, m), 1.38–1.63 (5H, m), 1.14–1.36 (3H, m); LCMS (high pH) (M + H)^+^ = 462.4, *R*_t_ = 0.80 min (100%).

#### (*S*)-5-(1-((1-(1-Isopropylpiperidine-4-carbonyl)piperidin-3-yl)methyl)-1*H*-benzo [*d*]imidazole-2-yl)-1,3-dimethylpyridin-2(1*H*)-one (31)

DIPEA (39 μL, 0.22 mmol) was added to a solution of 1-*iso*propylpiperidine-4-carboxylic acid (13 mg, 0.08 mmol) and HATU (35 mg, 0.09 mmol) in DMF (0.5 mL) at rt. Following stirring at rt for 10 min, a solution of **19** (25 mg, 0.07 mmol) in DMF (0.5 mL) was added. The reaction mixture was stirred at rt for 1 h, and then CH_2_Cl_2_ (20 mL) and 10% aq LiCl (20 mL) were added. The separated aqueous phase was extracted with 4:1 CHCl_3_/IPA (2 × 20 mL). The combined organic phase was washed with 10% aq LiCl (3 × 20 mL), passed through a hydrophobic frit, and evaporated under reduced pressure. The resultant residue was dissolved in 1:1 MeOH/DMSO (1 mL) and purified by preparative HPLC, eluting with 15–100% MeCN/10 mM aq ammonium bicarbonate. The appropriate fractions were combined and extracted with 4:1 CHCl_3_/IPA (2 × 20 mL). The combined organic phase was passed through a hydrophobic frit and evaporated under reduced pressure to give **31** as a pale-yellow solid (21 mg, 58%). ^1^H NMR (400 MHz, 393 K, DMSO-*d*_6_) δ 7.98 (1H, d, *J* = 2.4 Hz), 7.61–7.68 (2H, m), 7.56–7.60 (1H, m), 7.20–7.29 (2H, m), 4.20–4.32 (2H, m), 3.92 (1H, br. d, *J* = 12.2 Hz), 3.58–3.67 (4H, m), 2.61–2.84 (5H, m), 2.13–2.23 (4H, m), 1.88–2.07 (3H, m), 1.39–1.63 (5H, m), 1.14–1.37 (3H, m), 1.09 (6H, d, *J* = 5.9 Hz); ^13^C NMR (100.6 MHz, DMSO-*d*_6_) δ *additional peaks due to rotamers* 172.6, 172.0, 161.7, 150.4, 150.3, 142.4, 138.3, 136.7, 136.6, 135.8, 127.9, 127.8, 122.2, 121.9, 118.8, 111.2, 111.0, 108.1, 108.0, 62.0, 53.7, 47.9, 47.6, 47.5, 47.3, 46.8, 46.7, 45.1, 44.3, 41.4, 40.4, 40.3, 40.2, 40.1, 40.0, 38.1, 38.0, 37.8, 37.3, 35.8, 28.9, 28.8, 28.6, 28.0, 27.3, 25.4, 24.6, 24.2, 18.0, 17.8, 16.8; HRMS (M + H)^+^ exact mass calculated for C_29_H_40_N_5_O_2_ 490.3183, found 490.3177; LCMS (high pH) (M + H)^+^ = 490.4, *R*_t_ = 0.88 min (100%).

#### (*S*)-1,3-Dimethyl-5-(1-((1-(tetrahydro-2*H*-pyran-4-carbonyl)piperidin-3-yl)methyl)-1*H*-benzo[*d*]imidazole-2-yl)pyridin-2(1*H*)-one (**32**)

DIPEA (78 μL, 0.45 mmol) was added to a stirred solution of tetrahydro–2*H*–pyran–4–carboxylic acid (19 mg, 0.15 mmol) and HATU (71 mg, 0.19 mmol) in DMF (0.5 mL) at rt. Following stirring at rt for 10 min, a solution of **19** (50 mg, 0.15 mmol) in DMF (0.5 mL) was added. The reaction mixture was stirred at rt for 1 h, and then 4:1 CHCl_3_/IPA (20 mL) and 5% aq LiCl (20 mL) were added. The separated aqueous phase was extracted with 4:1 CHCl_3_/IPA (2 × 20 mL). The combined organic phase was washed with brine (2 × 20 mL), passed through a hydrophobic frit, and evaporated under reduced pressure. The resultant residue was dissolved in 1:1 MeOH/DMSO (1 mL) and purified by MDAP (high pH). The appropriate fractions were combined and extracted with CH_2_Cl_2_ (2 × 40 mL). The combined organic phase was passed through a hydrophobic frit and evaporated under reduced pressure to give **32** as a yellow solid (29 mg, 44%). ^1^H NMR (400 MHz, 393 K, DMSO-*d*_6_) δ 7.96 (1H, d, *J* = 2.2 Hz), 7.60–7.65 (2H, m), 7.53–7.58 (1H, m), 7.17–7.26 (2H, m), 4.17–4.30 (2H, m), 3.89 (1H, br. d, *J* = 12.7 Hz), 3.68–3.80 (2H, m), 3.64 (1H, br. d, *J* = 12.0 Hz), 3.56 (3H, s), 3.14–3.29 (2H, m), 2.74–2.85 (1H, integrates for 6H due to the overlapping water peak, m), 2.67 (1H, dd, *J* = 13.0, 10.0 Hz), 2.44–2.56 (1H, integrates for 9H due to the overlapping DMSO peak, m), 2.11 (3H, s), 1.85–1.98 (1H, m), 1.42–1.62 (4H, m), 1.33–1.41 (1H, m), 1.11–1.29 (3H, m); LCMS (high pH) (M + H)^+^ = 449.3, *R*_t_ = 0.79 min (99%).

#### (*S*)-5-(1-((1-(1-Acetylpiperidine-4-carbonyl)piperidin-3-yl)methyl)-1*H*-benzo[*d*]imidazole-2-yl)-1,3-dimethylpyridin-2(1*H*)-one (**33**)

DIPEA (39 μL, 0.22 mmol) was added to a solution of 1-acetylpiperidine-4-carboxylic acid (13 mg, 0.07 mmol) and HATU (35 mg, 0.09 mmol) in DMF (0.5 mL) at rt. Following stirring at rt for 10 min, a solution of **19** (25 mg, 0.07 mmol) in DMF (0.5 mL) was added. The reaction mixture was stirred at rt for 66 h, and then CH_2_Cl_2_ (20 mL) and 10% aq LiCl (20 mL) were added. The separated aqueous phase was extracted with CH_2_Cl_2_ (20 mL). The combined organic phase was washed with 10% aq LiCl (3 × 20 mL), passed through a hydrophobic frit, and evaporated under reduced pressure. The resultant residue was dissolved in 1:1 MeOH/DMSO (1 mL) and purified by preparative HPLC, eluting with 15–100% MeCN/10 mM aq ammonium bicarbonate. The appropriate fractions were combined and extracted with CH_2_Cl_2_/IPA (2 × 20 mL). The combined organic phase was passed through a hydrophobic frit and evaporated under reduced pressure to give **33** as a white solid (19 mg, 52%). ^1^H NMR (400 MHz, 393 K, DMSO-*d*_6_) δ 7.99 (1H, d, *J* = 2.2 Hz), 7.62–7.68 (2H, m), 7.57–7.61 (1H, m), 7.21–7.30 (2H, m), 4.21–4.33 (2H, m), 3.84–4.06 (3H, m), 3.69 (1H, br. d, *J* = 12.2 Hz), 3.59 (3H, s), 2.66–2.91 (3H, integrates as 8H due to the overlapping water peak, m), 2.53–2.62 (1H, m), 2.14 (3H, s), 1.87–2.00 (4H, m), 1.14–1.65 (9H, m); LCMS (high pH) (M + H)^+^ = 490.4, *R*_t_ = 0.76 min (100%).

#### (*S*)-5-(1-((1-(1-(2-Fluoroethyl)piperidine-4-carbonyl)piperidin-3-yl)methyl)-1*H*-benzo[*d*]imidazole-2-yl)-1,3-dimethylpyridin-2(1*H*)-one (**34**)

1-Bromo-2-fluoroethane (13 μL, 0.18 mmol) was added to a stirring solution of NaH (60% by wt dispersion in oil) (7 mg, 0.18 mmol) and **27** (54 mg, 0.12 mmol) in DMF (1 mL) at rt under N_2_. The reaction mixture was stirred at rt under N_2_ for 30 min, and then additional NaH (60% by wt dispersion in oil) (7 mg, 0.18 mmol) and 1-bromo-2-fluoroethane (13 μL, 0.18 mmol) were added. The reaction mixture was heated to 60 °C for 1 h and then allowed to cool to rt. MeOH (1 mL) was added, and the mixture was concentrated under reduced pressure. The resultant residue was dissolved in 1:1 MeOH/DMSO (2 mL) and purified by preparative HPLC, eluting with 0–100% MeCN/10 mM aq ammonium bicarbonate. The appropriate fractions were combined and extracted with CH_2_Cl_2_ (2 × 20 mL). The combined organic phase was passed through a hydrophobic frit and evaporated under reduced pressure to give **34** as a white solid (22 mg, 37%). ^1^H NMR (400 MHz, 393 K, DMSO-*d*_6_) δ 7.97–8.00 (1H, m), 7.62–7.68 (2H, m), 7.56–7.60 (1H, m), 7.20–7.30 (2H, m), 4.53–4.57 (1H, m), 4.41–4.45 (1H, m), 4.21–4.33 (2H, m), 3.91 (1H, br. d, *J* = 12.5 Hz), 3.61–3.69 (1H, m), 3.59 (3H, s), 2.68–2.86 (2H, integrates as 8H due to the overlapping water peak, m), 2.63–2.68 (1H, m), 2.56–2.61 (1H, m), 2.18–2.29 (1H, m), 2.14 (3H, s), 1.87–2.07 (3H, m), 1.40–1.64 (6H, m), 1.14–1.38 (4H, m); LCMS (high pH) (M + H)^+^ = 494.4, *R*_t_ = 0.84 min (100%).

#### (*S*)-5-(1-((1-(1-(2,2-Difluoroethyl)piperidine-4-carbonyl)piperidin-3-yl)methyl)-1*H*-benzo[*d*]imidazole-2-yl)-1,3-dimethylpyridin-2(1*H*)-one (**35**)

2-Bromo-1,1-difluoroethane (27 μL, 0.35 mmol) was added to a stirring solution of NaH (60% by wt dispersion in oil) (14 mg, 0.35 mmol) and **27** (103 mg, 0.20 mmol) in DMF (2 mL) at rt under N_2_. The reaction mixture was stirred at 60 °C under N_2_ for 16 h. Upon cooling to rt, additional NaH (60% by wt dispersion in oil) (14 mg, 0.35 mmol) and 2-bromo-1,1-difluoroethane (27 μL, 0.35 mmol) were added, and the mixture was heated to 70 °C for 1 h. Additional 2-bromo-1,1-difluoroethane (54 μL, 0.69 mmol) was added, and the reaction mixture was stirred at 80 °C for 2 h and then 90 °C for 18 h. Upon cooling to rt, MeOH (2 mL) was added, and the mixture evaporated under reduced pressure. The resultant residue was dissolved in 1:1 MeOH/DMSO (2 mL) and purified by preparative HPLC, eluting with 0–100% MeCN/10 mM aq ammonium bicarbonate. The appropriate fractions were combined and extracted with CH_2_Cl_2_ (2 × 20 mL). The combined organic phase was passed through a hydrophobic frit and evaporated under reduced pressure to give compound **35** as a white solid (44 mg, 37%). ^1^H NMR (400 MHz, 393 K, DMSO-*d*_6_) δ 7.98 (1H, d, *J* = 2.4 Hz), 7.61–7.68 (2H, m), 7.56–7.60 (1H, m), 7.20–7.30 (2H, m), 5.98 (1H, tt, *J* = 55.5, 3.9 Hz), 4.20–4.32 (2H, m), 3.90 (1H, br. d, *J* = 13.2 Hz), 3.65 (1H, br. d, *J* = 11.2 Hz), 3.59 (3H, s), 2.65–2.87 (4H, integrates as 10H due to the overlapping water peak, m), 2.06–2.32 (6H, m), 1.89–1.98 (1H, m), 1.40–1.64 (7H, m), 1.11–1.37 (4H, m); LCMS (high pH) (M + H)^+^ = 512.3, *R*_t_ = 0.88 min (100%).

#### (*S*)-1,3-Dimethyl-5-(1-((1-(1-(2,2,2-trifluoroethyl)piperidine-4-carbonyl)piperidin-3-yl)methyl)-1*H*-benzo[*d*]imidazole-2-yl)pyridin-2(1*H*)-one (**36**)

Phenylsilane (13 μL, 0.11 mmol) and TFA (7 μL, 0.09 mmol) were added to a solution of **27** (23 mg, 0.05 mmol) in THF (0.2 mL) at rt. The vial was sealed, and the reaction mixture was heated at 70 °C for 17 h. Additional phenylsilane (13 μL, 0.11 mmol) and TFA (7 μL, 0.09 mmol) were added, and the reaction mixture was reheated at 70 °C for 2 h. Following cooling to rt, the reaction mixture was evaporated under reduced pressure. The resultant residue was dissolved in 1:1 MeOH/DMSO (1 mL) and purified by preparative HPLC, eluting with 15–100% MeCN/10 mM aq ammonium bicarbonate. The appropriate fractions were combined and extracted with CH_2_Cl_2_ (2 × 20 mL). The combined organic phase was passed through a hydrophobic frit and evaporated under reduced pressure to give **36** as a white solid (12 mg, 44%). ^1^H NMR (400 MHz, 393 K, DMSO-*d*_6_) δ 7.98 (1H, d, *J* = 2.4 Hz), 7.62–7.68 (2H, m), 7.56–7.60 (1H, m), 7.20–7.30 (2H, m), 4.20–4.33 (2H, m), 3.90 (1H, br d, *J* = 12.7 Hz), 3.57–3.69 (4H, m), 3.07 (2H, q, *J* = 10.3 Hz), 2.77–2.91 (3H, integrates as 10H due to the overlapping water peak, m), 2.72 (1H, dd, *J* = 13.4, 10.0 Hz), 2.21–2.36 (3H, m), 2.14 (3H, s), 1.87–2.00 (1H, m), 1.40–1.65 (5H, m), 1.14–1.39 (3H, m); LCMS (high pH) (M + H)^+^ = 530.5, *R*_t_ = 0.97 min (100%).

### Computational Methods

For protein preparation, ligand preparation, docking studies, and WaterMap simulations, the OPLS-3e force field was used throughout.^56^

### Complex Preparation

The apo structure of BRD4 BD1 (pdb: 2oss) and the bound structure of **14** in BRD2 BD2 (pdb: 8px8) were prepared with the Protein Preparation Wizard tool in Maestro Release 2018.04. Bond orders were assigned to the ligands, and hydrogens were added. The hydrogen bonding network was optimized with the ProtAssign algorithm at a neutral pH such that the amide group of Asn, and Gln residues, the thiol and hydroxyl groups, and the imidazole rings of His residues were adjusted to the environment. Restrained minimization using the Impref module of Impact and the OPLS-3e force field was performed for refinement of the structures. This minimization continued until the average RMSD of the non-hydrogen atoms reached the specified limit of 0.3 Å.

### Metadynamics

The prepared complexes of apo BRD4-BD2 (pdb: 2ouo) were solvated with TIP3P water molecules in a box of 10 × 10 × 10 Å. Ions were added to neutralize the system and reach a concentration of 0.15 M. The prepared complexes were then submitted to the metadynamics simulations with Desmond v5.6, using as CVs the χ1 and χ2 torsional angles of His433. Gaussians were deposited every 0.09 ps with a hill height of 0.03 kcal/mol. The width of the Gaussian was set to 5°. The metadynamics simulation lasted less than 100 ns and was replicated three times, changing the seed to assign starting velocities. The evaluation of the convergence was based on three criteria. First, the FES was compared through an interval of 10 ns, and to consider a simulation convergence, the free-energy profile should not change significantly. This was achieved by comparing the FES at different simulation times. Second, the evolution of each CV was monitored so that they were not trapped in specific energy minima. Third, it was checked that the FES profiles of different replicas were superposable. The FES were calculated by using 65, 218, and 171 ns. The block average was calculated every 5, 10, and 20 ns, respectively, to have a total of 12 FES to compare.

### Docking Calculations

(*S*)-**12** was prepared with the LigPrep module to determine the 3D structure and ionization state at pH 7.0 ± 2 in Maestro Release 2018.04. The Glide molecular docking tool was used to generate binding modes for (*S*)-**12** in BRD4 BD1.^57^ The bound ligands were selected to define the binding site. The four water molecules at the bottom of the pocket were retained during the preparation step. Default van der Waals scaling (1.0 for the receptor and 0.8 for the ligand) was used. Under the Advanced Settings, it was specified to use enhanced sampling for conformer generations and expanded sampling for the selection of initial poses. It was selected to keep at least 4 poses per ligand. The docked complexes of (*S*)-**12** were then visually inspected, and the poses that satisfied the H-bond interaction with Asn140 and inserting the acetyl group in the Asp144 cavity were selected and used for WaterMap simulations.

### WaterMap

The WaterMap calculations were run with the default parameters using BRD4 BD1 apo protein (pdb: 2oss) and the docked solutions of (*S*)-**12** in the BRD4 BD1 domain appropriately prepared as described above. From WaterMap, the location, occupancy, enthalpy, entropy, and free energy of the water molecules were calculated by using a combination of molecular dynamics, solvent clustering, and statistical thermodynamics analysis. For the definition of the binding site, the ligand was selected and retained for the 2.0 ns long molecular dynamics simulation with the OPLS3e force field; in the case of the apo protein, the residues Asn140, Asp144, and Ile146 were selected as references for the binding site. Water sites around Asp144 were analyzed. The existing water molecules at the bottom of the pocket were retained and treated as solvents in the calculations. The coordinates of the protein were restrained with a 5.0 kcal/mol/Å^2^ harmonic potential on the heavy atoms to ensure convergence of the water sampling around the protein conformation. The frames from the molecular dynamics simulation were then spatially clustered to form localized hydration sites.

### BioMAP Phenotypic Profile

BioMAP Diversity PLUS coculture assays (Eurofins DiscoverX, South San Francisco, CA) were performed as previously described.^58^**31** was tested at a range of concentrations (10 μM, 2.5 μM, 630 nM, 160 nM) in 0.1% DMSO. **31** was added 1 h before stimulation of the cells and was present during the entire incubation period of the assays.

### *In Vitro* Assays

All TR-FRET and hWB cytokine assays have been described previously.^54^

## Supporting Information Available

All screening statistics, full BROMO*scan* and selectivity data, ^1^H and ^13^C NMR spectra for **31**, representative LCMS traces of target compounds, and X-ray data collection and refinement statistics (PDF)
Molecular formula strings (CSV)
Docking of compound **12** into BRD4 BD1 (PDB)

## Supplementary Material

jm3c00906_si_002.csv

jm3c00906_si_003.pdb

## Accession Codes

Coordinates have been deposited with the Protein Data Bank under accession codes 8px2 (BRD2 BD2/**13** complex), 8px8 (BRD2 BD2/**14** complex), and 8pxa (BRD4 BD1/**31** complex). The authors will release atomic coordinates and experimental data upon article publication.

## Abbreviations

aq: aqueous
BD1: bromodomain 1 (N-terminal bromodomain)
BD2: bromodomain 2 (C-terminal bromodomain)
BRD2: bromodomain containing protein 2
BRD3: bromodomain containing protein 3
BRD4: bromodomain containing protein 4
BRDT: bromodomain containing protein, testis-specific
CAD: charged aerosol detection
CLND: chemiluminescent nitrogen detection
CV: collective variables
EP300: E1A-associated protein p300
FES: free energy surface
hWB: human whole blood
LCMS: liquid chromatography mass spectrometry
LE: ligand efficiency
LipE: lipophilic efficiency
MCP-1: monocyte chemoattractant protein-1
MDAP: mass-directed auto preparation
pIC_50_: –log_10_(IC_50_)
pdb: protein data bank
TR-FRET: time-resolved Fürster resonance energy transfer
WPF: tryptophan-proline-phenylalanine
