# Supplementary material for: Structure-Guided Design of a Domain-Selective Bromodomain and Extra Terminal N-Terminal Bromodomain Chemical Probe
Source: J Med Chem. 2023 Nov 15;66(23):15728–49. doi: 10.1021/acs.jmedchem.3c00906 (PMC10726358; doi:10.1021/acs.jmedchem.3c00906)
Supplement: Supplementary file 1 — jm3c00906_si_001.pdf [file jm3c00906_si_001.pdf]

Supporting Information

Structure Guided Design of a Domain Selective Bromodomain and Extra Terminal (BET) N-Terminal Bromodomain Chemical Probe

Erin Bradley,[†,‡] Lucia Fusani,[†,‡] Chun-wa Chung,[†] Peter D. Craggs,[†] Emmanuel H. Demont,[†] Philip G. Humphreys,[†] Darren J. Mitchell,[†] Alex Phillipou,[†] Inmaculada Rioja,[†] Rishi R. Shah,[†] Christopher R. Wellaway,[†] Rab K. Prinjha,[†] David S. Palmer,[‡] William J. Kerr,[‡]* Marc Reid,[‡] Ian D. Wall,[†] Rosa Cookson[†]**

[†]GSK, Medicines Research Centre, Stevenage, Hertfordshire, SG1 2NY, United Kingdom

[‡]Department of Pure and Applied Chemistry, University of Strathclyde, Thomas Graham Building, 295 Cathedral Street, Glasgow, G1 1XL, United Kingdom

*Corresponding Author: E-mail address: philip.g.humphreys@gsk.com; r.cookson@celeristx.com; w.kerr@strath.ac.uk

Table of contents

Table S1, Full data table for exemplified compounds S2
Table S2, BET family TR-FRET data for **31** S3
Table S3, BROMO*scan* selectivity data for **31** S4
Table S4, Cross screening data for **31** S5
^{1}H NMR and ^{13}C NMR spectra for **31** S6
LCMS traces of key compounds S7
Crystallization and crystallography materials S12
References S15

Compound	BRD4 BD1 pIC_{50}	BRD4 BD2 pIC_{50}	hWB MCP-1 pIC_{50}	Chrom Log$D_{pH7.4}$	CAD (µg/mL)	CLND (µg/mL)
**(R)-12**	7.5 ± 0.26 (9)	6.3 ± 0.13 (10)	-	2.3	-	≥ 169
**(S)-12**	8.0 ± 0.11 (2)	6.2 ± 0.06 (3)	-	2.2	-	≥ 247
**13**	7.2 ± 0.13 (10)	6.4 ± 0.12 (10)	-	2.7	-	≥ 142
**14**	8.0 ± 0.06 (2)	6.3 ± 0.01 (2)	-	3.3		≥ 250
**19**	6.2 ± 0.14 (2)	5.0 ± 0.09 (2)	-	0.7		≥ 152
**21**	7.8 ± 0.17 (4)	5.6 ± 0.06 (4)	6.3 ± 0.34 (2)	1.2	113	-
**22**	8.0 ± 0.25 (3)	5.9 ± 0.08 (3)	6.2 ± 0.07 (4)	1.2	≥ 169	-
**23**	7.9 ± 0.14 (3)	5.4 ± 0.05 (3)	6.7 ± 0.09 (3)	1.2	≥ 230	-
**24**	7.6 ± 0.16 (3)	5.3 ± 0.06 (3)	6.0 ± 0.03 (4)	1.3	≥ 208	-
**25**	7.7 ± 0.09 (3)	5.0 ± 0.03 (3)	6.0 ± 0.11 (3)	1.4	≥ 157	-
**26**	7.0 ± 0.04 (3)	4.9 ± 0.07 (2)	5.6 (1)	1.9	≥ 160	-
**27**	7.5 ± 0.07 (4)	4.9 ± 0.1 (4)	6.0 ± 0.19 (3)	1.3	≥ 215	-
**28**	7.9 ± 0.1 (2)	5.1 ± 0.03 (2)	6.5 ± 0.12 (3)	1.4	≥ 155	-
**29**	6.8 ± 0.07 (3)	5.1 ± 0.06 (3)	5.8 (1)	1.7	≥ 204	-
**30**	7.6 ± 0.17 (9)	4.8 ± 0.13 (9)	6.6 ± 0.18 (73)	1.5	≥ 237	-
**31**	7.8 ± 0.17 (9)	4.8 ± 0.15 (9)	6.9 ± 0.61 (8)	1.7	≥ 257	-
**32**	6.7 ± 0.07 (3)	4.7 ± 0.26 (3)	6.1 ± 0.11 (4)	2.7	≥ 245	-
**33**	6.5 ± 0.11 (3)	4.7 ± 0.15 (3)	5.7 ± 0.21 (3)	2.2	≥ 205	-
**34**	7.6 ± 0.04 (3)	4.7 ± 0.18 (3)	7.2 ± 0.09 (3)	2.5	≥ 207	-
**35**	6.7 ± 0.09 (3)	4.7 ± 0.2 (3)	6.2 ± 0.08 (2)	3.2	≥ 279	-
**36**	6.3 ± 0.04 (3)	4.7 ± 0.14 (3)	5.9 ± 0.21 (4)	4.2	≥ 201	-

Table S1. Full biological profiling from Tables 1 and 2, including mean pIC_{50}, standard deviation, and the number of test occasions.

Compound	31
**BRD2 BD1 pIC$_{50}$**	8.0 ± 0.21 (2)
**BRD2 BD2 pIC$_{50}$**	5.4 (1)
**BRD3 BD1 pIC$_{50}$**	7.7 ± 0.02 (2)
**BRD3 BD2 pIC$_{50}$**	5.1 ± 0.08 (2)
**BRD4 BD1 pIC$_{50}$**	7.8 ± 0.17 (9)
**BRD4 BD2 pIC$_{50}$**	4.8 ± 0.15 (9)
**BRDT BD1 pIC$_{50}$**	7.3 ± 0.12 (2)
**BRDT BD2 pIC$_{50}$**	4.5 ± 0.01 (2)

Table S2. Full biological profiling from Table 3, including mean pIC$_{50}$, standard deviation, and the number of test occasions.

Bromodomain	31<br>pKd
BRD2 BD1	8.5
BRD2 BD2	5.5
BRD3 BD1	8.4
BRD3 BD2	5.8
BRD4 BD1	8.1
BRD4 BD2	5.6
BRDT BD1	8.1
BRDT BD2	5.5
EP300	5.0
BRPF1	4.8
CREBBP	4.7
BRD9	4.6
ATAD2A	<4.5
ATAD2B	<4.5
BAZ2A	<4.5
BAZ2B	<4.5
BRD1	<4.5
BRD7	<4.5
BRD8 BD1	<4.5
BRD8 BD2	<4.5
BRPF3	<4.5
CECR2	<4.5
FALZ	<4.5
GCN5	<4.5
PBRM1 BD2	<4.5
PBRM1 BD5	<4.5
PCAF	<4.5
SMARCA2	<4.5
SMARCA4	<4.5

Table S3. Selectivity profile of **31** in the BROMO*scan* panel (DiscoveRx Corp)

Assay	31 pXC_{50}
Alpha 2C Adrenoceptor agonist	<4.0 (2)
5-HT1B Agonist	<4.0 (2)
Adenosine A2a Human Agonist	<4.0 (2)
Beta 2 Adrenoceptor Human Agonist	<4.0 (2)
K1 Agonist	<4.0 (2)
5-HT3 Agonist	<4.3 (2)
Dopamine D2 Agonist	<4.0 (2)
M2 (CHRM2) Human Ag	<4.3 (3)
CB1 Agonist	<4.0 (2)
MRGPRX2 Agonist	<4.0 (2)
M1 Agonist	<4.3 (2)
5-HT2A Agonist	<4.0 (2)
5-HT2B Agonist	<4.0 (2)
5-HT2C Agonist	<4.0 (2)
Aryl Hydrocarbon Receptor	<4.0 (2)
AR Agonist	<4.0 (2)
Alpha 1 nAChR Agonist	<4.2 (4)
Alpha 1 nAChR Antagonist	<4.2 (4)
GR Agonist	<4.0 (2)
OPRK1 Agonist	<4.0 (2)
OPRM1 Agonist	<4.0 (2)
PXR Human Agonist	<4.3 (2)
Kv1.5 (KCNA5) blocker	<4.3 (2)
Dopamine 2 (D2) Antagonist	<4.0 (2)
Cyclooxygenase 2 (COX-2) antagonist	<4.0 (2)
OATP1B1 inhibitor	<4.3 (2)
Acetylcholinesterase (AChE) antagonist	<4.0 (2)
NET blocker	<4.0 (2)
hCav1_2 Blocker	<4.0 (2)
LCK inhibitor	<4.5 (2)
Beta 2 Adrenoceptor Antagonist	<4.0 (2)
PI3K-gamma inhibitor	<4.5 (1)
PDE4B antagonist	<4.0 (2)
SERT blocker	<4.0 (2)
5-HT3 antagonist	<4.3 (2)
NMDA 1A/2B antagonist	<4.0 (2)
CYP3A4 inhibition	4.1 (2)
Monoamine Oxidase A antagonist	<4.0 (2)
Aurora B inhibitor	<4.5 (2)
M2 (CHRM2) antagonist	<4.3 (4)
BSEP inhibitor	<3.7 (2)
D1 antagonist	<4.0 (2)
Alpha 1B antagonist	<4.6 (2)
Histamine 1 antagonist	<4.6 (2)
M1 antagonist	<4.3 (2)
NK1 antagonist	<4.6(2)
5-HT2A antagonist	<4.0 (2)
5-HT2C antagonist	<4.0 (2)
V1a antagonist	<4.3 (2)
Nav1.5 blocker	<4.3 (2)
hERG blocker	<4.3 (1)
KCNQ1 blocker	<4.6 (2)
Phospholipoidosis induction	<4 (3)

Table S4. Enhanced cross screening panel for **31**, including mean pXC_{50}, standard deviation, and the number of test occasions.

^{1}H NMR (400 MHz, 393 K, DMSO-d_6) and ^{13}C NMR (100.6 MHz, DMSO-d_6) spectra for 31

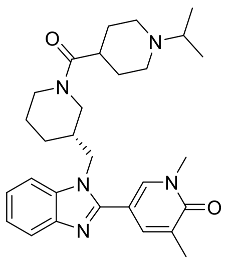

31

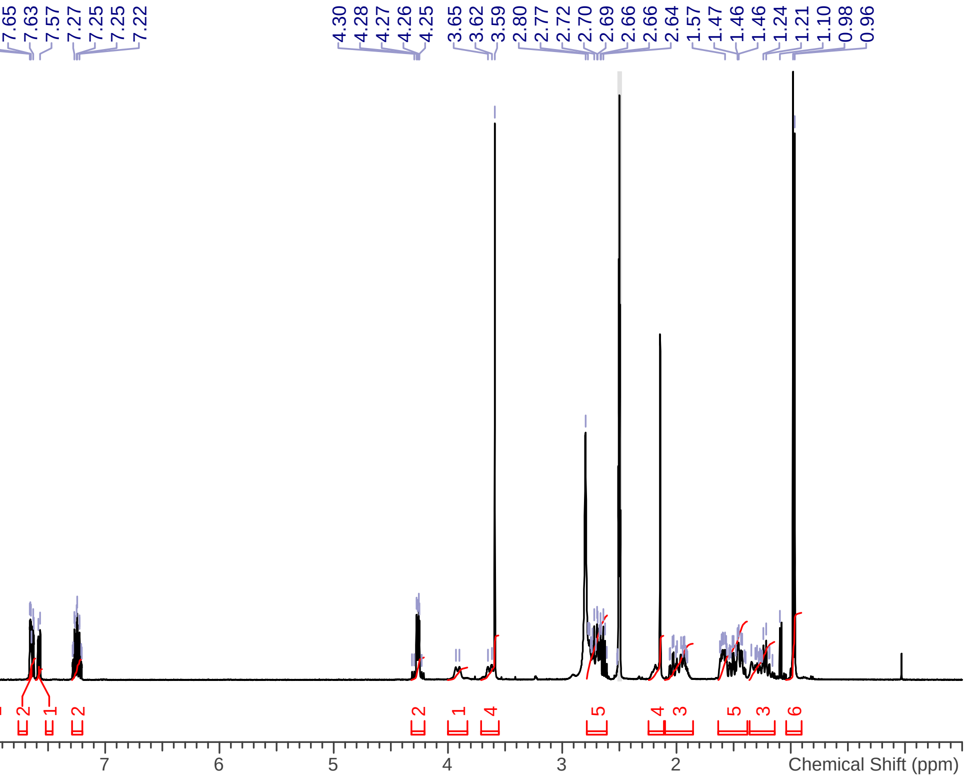

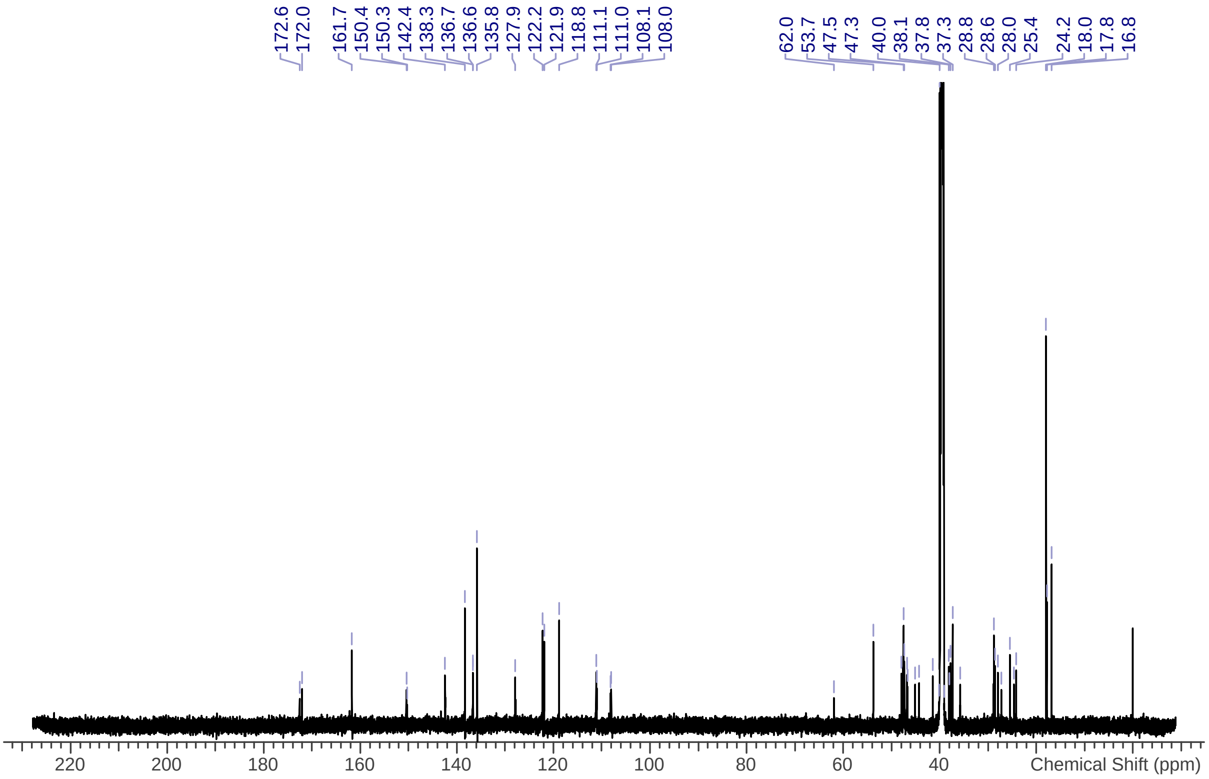

LCMS traces of key compounds

(R)-12

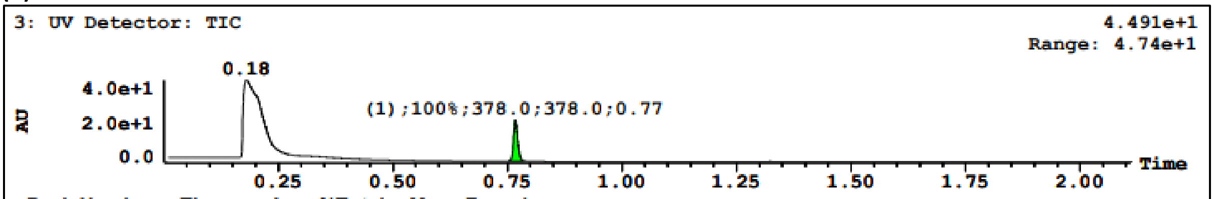

Peak Number	Time	Area %Total	Mass Found
1	0.77	100.00	378.00, 378.00

(S)-12

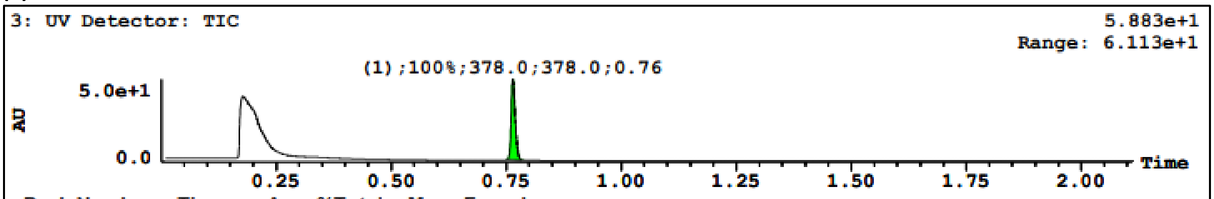

Peak Number	Time	Area %Total	Mass Found
1	0.76	100.00	378.00, 378.00

14

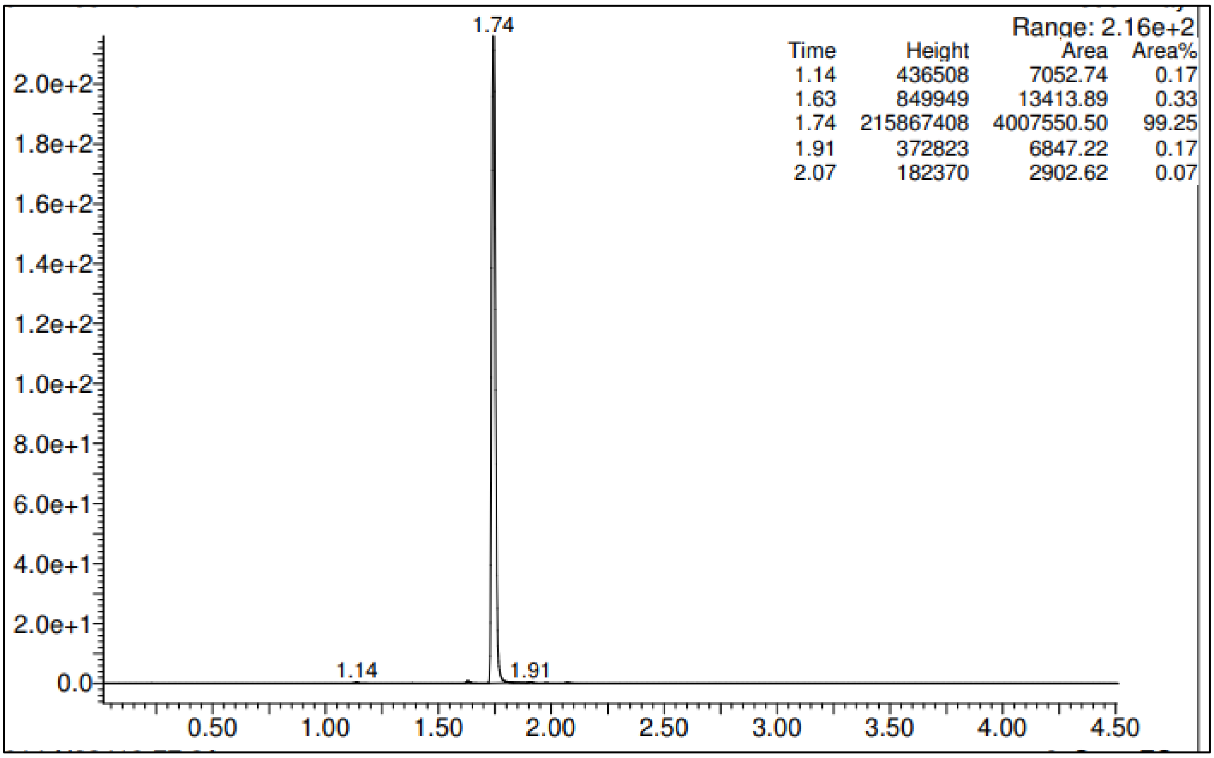

Time	Height	Area	Area%
1.14	436508	7052.74	0.17
1.63	849949	13413.89	0.33
1.74	215867408	4007550.50	99.25
1.91	372823	6847.22	0.17
2.07	182370	2902.62	0.07

19

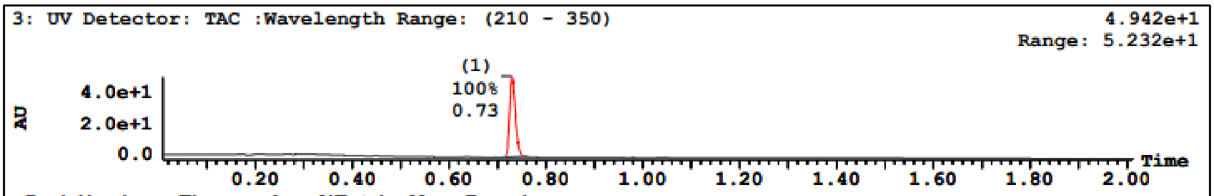

Peak Number	Time	Area %Total	Mass Found
1	0.73	100.00	Not Found

21

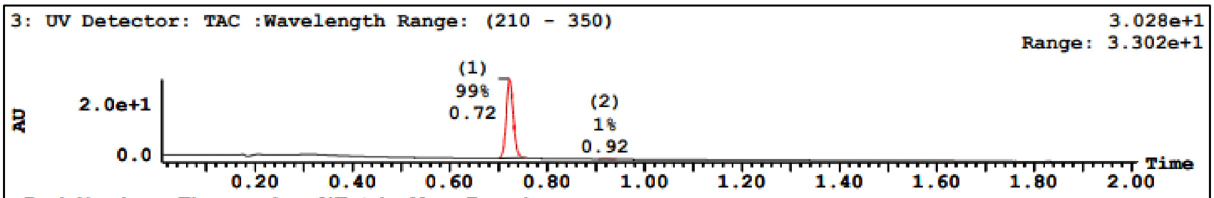

Peak Number	Time	Area %Total	Mass Found
1	0.72	98.83	Not Found
2	0.92	1.17	Not Found

22

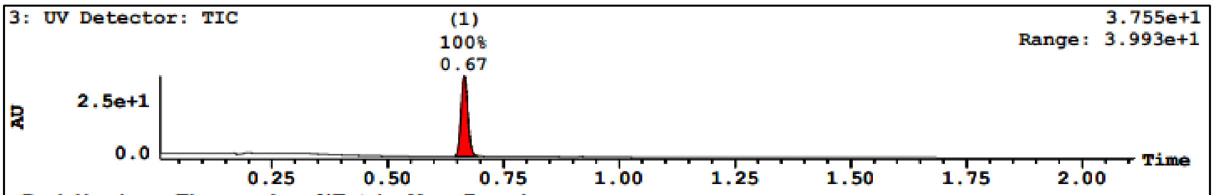

Peak Number	Time	Area %Total	Mass Found
1	0.67	100.00	

23

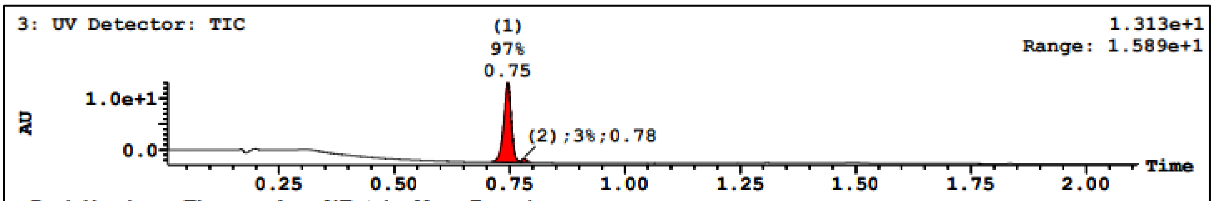

Peak Number	Time	Area %Total	Mass Found
1	0.75	97.05	
2	0.78	2.95	

24

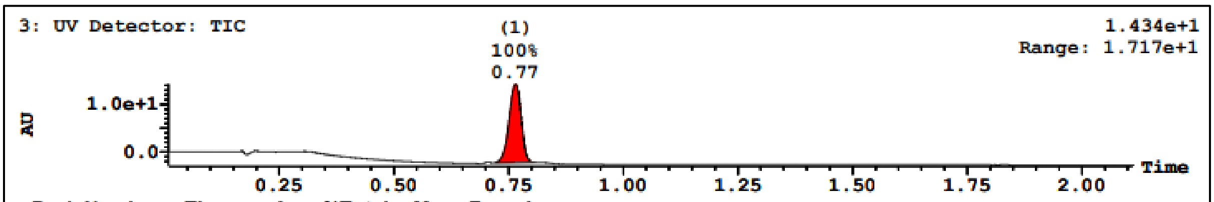

Peak Number	Time	Area %Total	Mass Found
1	0.77	100.00	

25

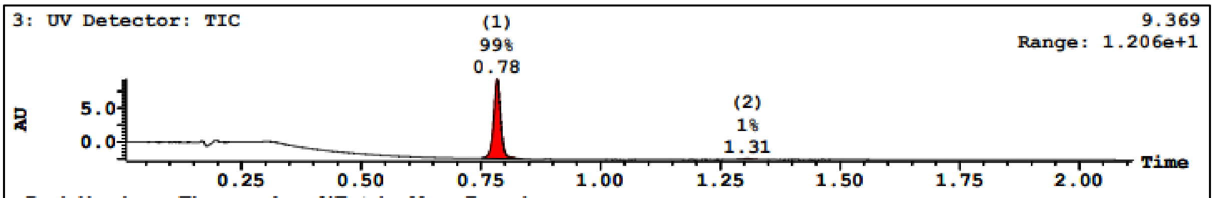

Peak Number	Time	Area %Total	Mass Found
1	0.78	98.83	
2	1.31	1.17	

26

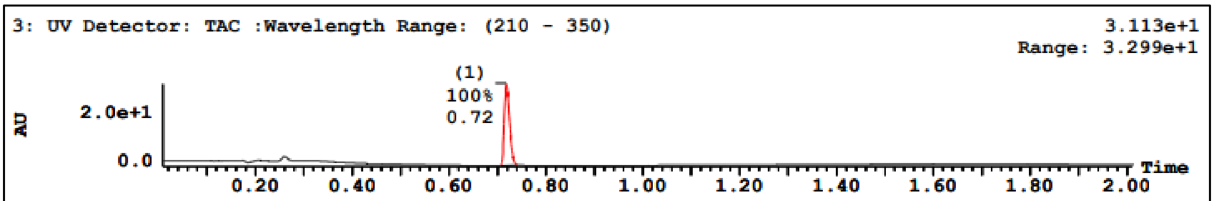

Peak Number	Time	Area %Total	Mass Found
1	0.72	100.00	Not Found

27

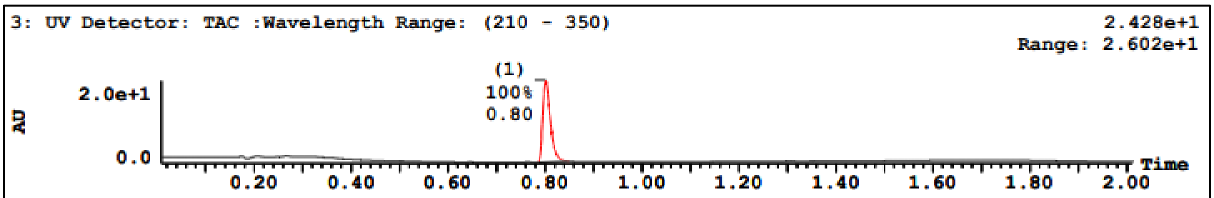

Peak Number	Time	Area %Total	Mass Found
1	0.80	100.00	Not Found

28

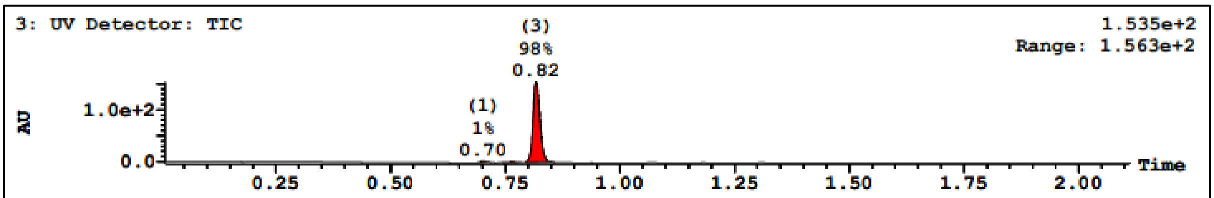

Peak Number	Time	Area %Total	Mass Found
1	0.70	0.99	
2	0.77	1.21	
3	0.82	97.80	

29

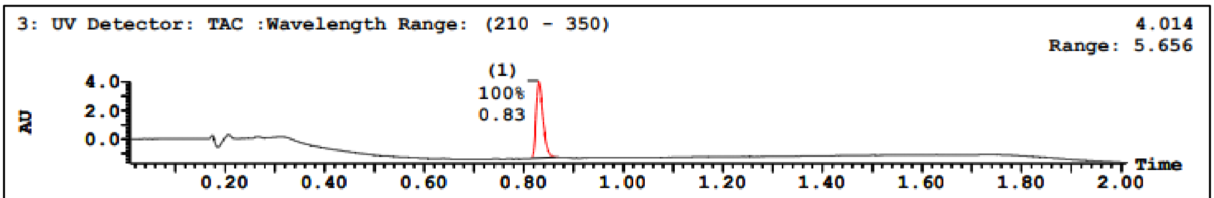

Peak Number	Time	Area %Total	Mass Found
1	0.83	100.00	Not Found

30

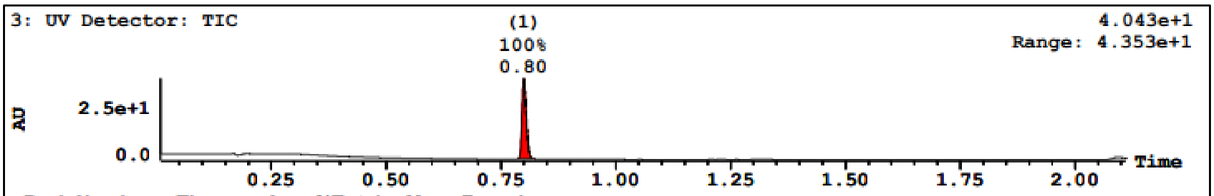

Peak Number	Time	Area %Total	Mass Found
1	0.80	100.00	

31

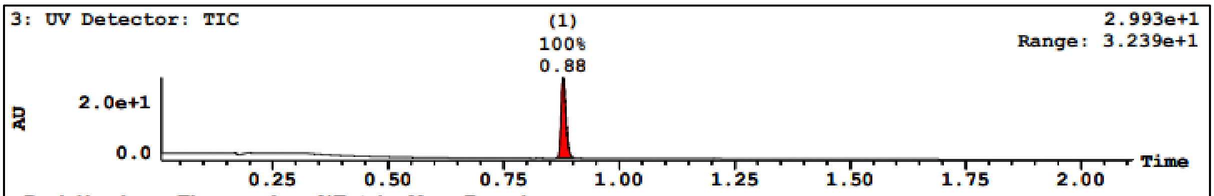

Peak Number	Time	Area %Total	Mass Found
1	0.88	100.00	

32

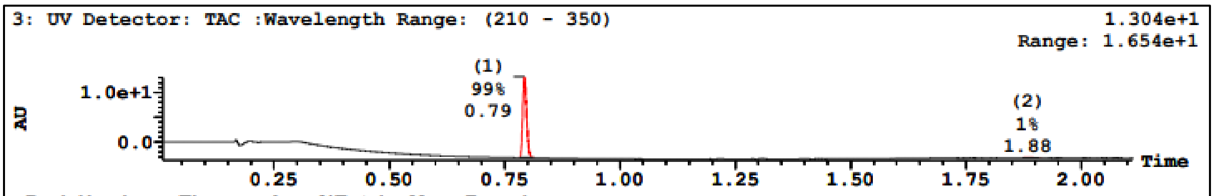

Peak Number	Time	Area %Total	Mass Found
1	0.79	98.60	Not Found
2	1.88	1.40	Not Found

33

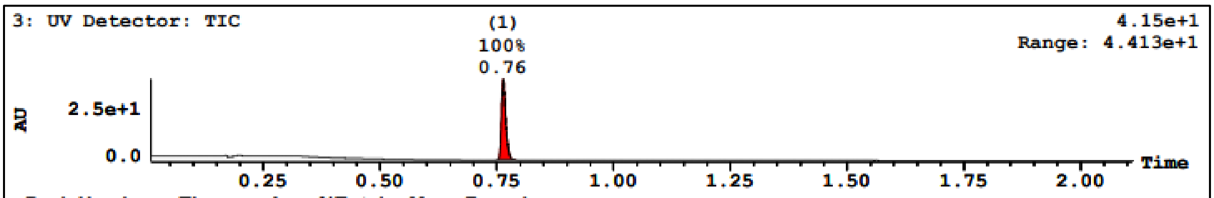

Peak Number	Time	Area %Total	Mass Found
1	0.76	100.00	

34

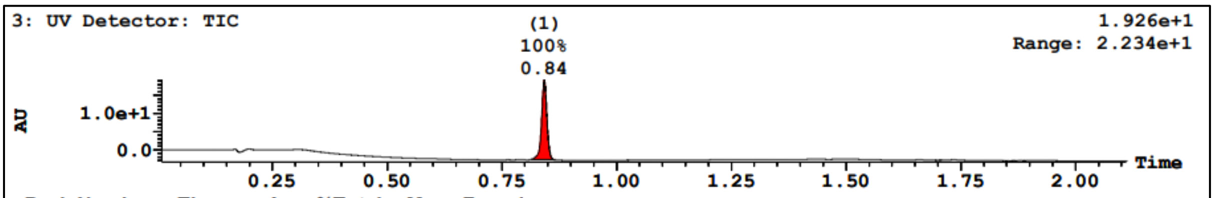

Peak Number	Time	Area %Total	Mass Found
1	0.84	100.00	

35

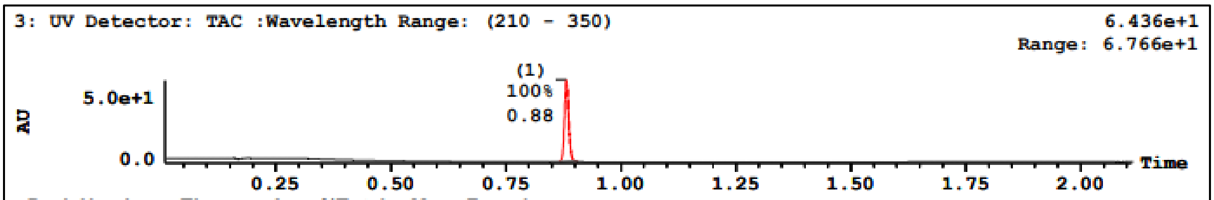

Peak Number	Time	Area %Total	Mass Found
1	0.88	100.00	Not Found

36

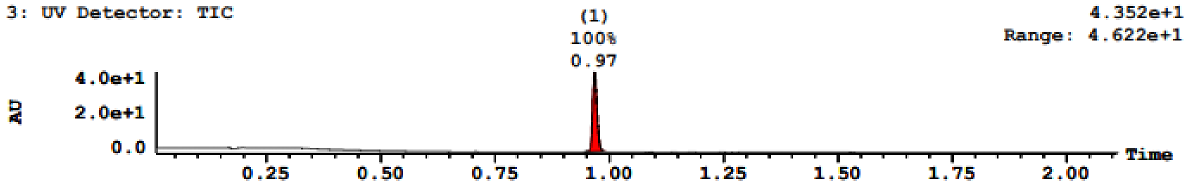

Peak Number	Time	Area %Total	Mass Found
1	0.97	100.00	

Crystallization and Crystallography Materials

All the statistics for the data collection and refined co-ordinates are given in Supplementary Table S5. The final crystal structures are deposited in the Protein Data Bank under these accession codes BRD2 BD2 / **13** (8px2), BRD2 BD2 / **14** (8px8) and BRD4 BD1 / **31** (8pxa).

BRD4-BD1 complex

1 × 200 µL protein aliquot was rapidly thawed. 1 µl of 2 mM of compound dissolved in DMSO was added to 50 µl protein in a new eppendorf and incubated on ice for ~1 h. The complex was centrifuged at 4 °C for 15 min at 13 krpm on a bench top centrifuge. 1:1 Drops of the 100 nL protein complex and 100 nL well solution were set up in sitting drops on Innovadyne SD2 96 well plates. 2 Diverse screens (PACT, Morpheus) were used for crystallisation. The crystallisation plates were stored at 20 °C in a Formulatrix imager. Data was collected from single crystal grown in 0.1 M Tris pH 8.5, 12% w/v 4K on at i04 at the DLS. The crystal was flash frozen directly from the drop in liquid nitrogen. Data was processed to 1.3 Å using XDS[1] and AIMLESS[2] within AutoPROC.[3] A molecular replacement solution was determined using Phaser[4] and a previously determined in house structure as a starting model. The $P2_12_12_1$ cell (a=b=g=90˚, a = 44.845 Å, b = 47.236 Å, c = 58.974 Å) has 1 molecule in the ASU. Manual model building was performed using COOT[4,5] and refined using REFMAC.[4,6] The difference density in the conserved acetylated lysine binding site could be unambiguously modelled by the ligand.

BRD2-BD2 complexes

BRD2-BD2 crystals were typically grown by streak seeding into hanging drops of 500 µL protein solution at 11-15 mg / mL and 500 µL well solution of 30% PEG 300, 0.1 M MES buffer pH 6.5 at 20°C. Crystals appeared within 24 h and were transferred into a fresh solution of the same well solution typically with 2% DMSO and compound at nominally >4 mM. Soaked crystals were briefly transferred into a solution consisting of 30% w/v PEG300, 0.1 M MES buffer, pH 6.5 supplemented with 10% ethylene glycol prior to flash freezing in liquid nitrogen. Data from a single crystal was collected 100 K on an in-house RIGAKU FR-E$^+$ SUPERBRIGHT/Saturn A200 detector / ACTOR robotic system and processed using XDS and aimless or mosflm and scala. [7] Manual model building was performed using COOT[4,5] and refined using REFMAC[6] within the CCP4 software suite.

Protein / compound	Fo-Fc OMIT map<br>**+1.0sigma (blue)**	2Fo-Fc OMIT map<br>**±3.0sigma (blue/red)**
**BRD2-BD2 / 13**<br>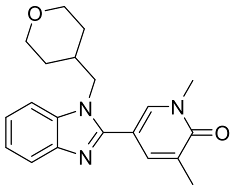	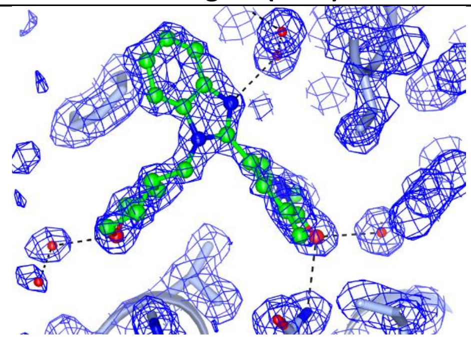	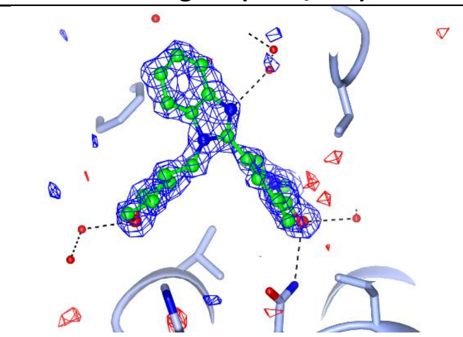
**BRD2-BD2 / 14**<br>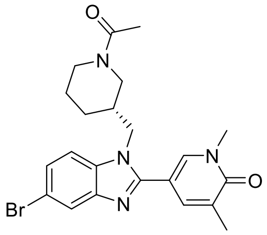	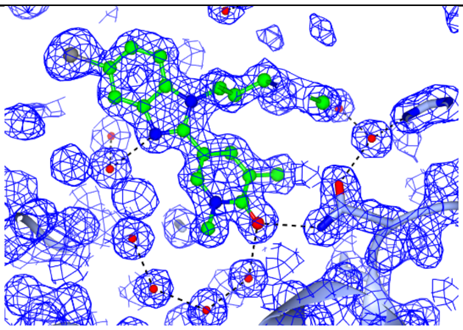	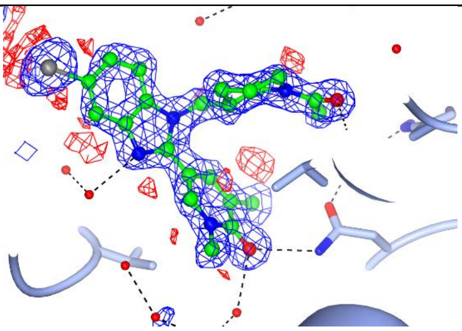
**BRD4-BD1 / 31**<br>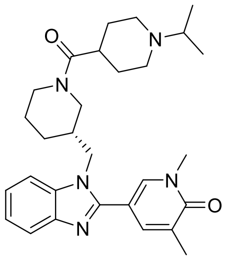	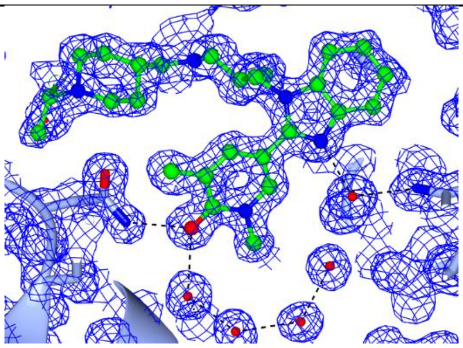	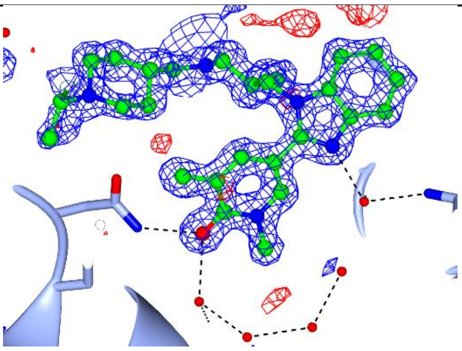

Figure S1. OMIT difference map (Fo-Fc) , after removal of the ligand from the final Xray structure model, is shown. The modelling of the binding mode of ligands are unambiguous with all elements of the molecule clearly defined.

(collection on a single crystal)	**BRD2-BD2 / 13**	**BRD2-BD2 / 14**	**BRD4-BD1 / 31**
**PDB code**	8px2	8px8	8pxa
**Data collection**			
Space group	$P2_12_12$	$P2_12_12$	$P2_12_12_1$
Cell dimensions			
a, *b*, *c* (Å)	71.949, 52.631, 32.159	71.825, 52.573, 31.975	44.845, 47.236, 58.974
α, β, γ (°)	90.000, 90.000, 90.000	90.000, 90.000, 90.000	90.00, 90.00, 90.00
Resolution (Å)	71.95-1.62 (1.71-1.62)	71.82-1.60 (1.69-1.60)	58.97-1.3 (1.37-1.30)
R_{merge}	0.024 (0.170)	0.018 (0.068)	0.060 (0.475)
ccI (1/2)	/	0.998 (0.839)	0.999 (0.751)
$I/\sigma I$	26.6 (5.6)	40.9 (12.2)	13.8 (1.9)
Completeness (%)	96.7 (83.6)	96.6 (81.0)	98.5(90.4)
Redundancy	2.7(1.9)	3.3 (2.0)	5.8 (2.9)
**Refinement**			
Resolution (Å)	71.95-1.62 (1.71-1.62)	71.82-1.60 (1.69-1.60)	58.97-1.3 (1.37-1.30)
No. reflections	41837 (3649)	52459 (3889	178538 (11814)
No. uniq reflections	15520 (1903)	15930 (1907)	31016 (4029)
$R_{work/}$ R_{free}	0.152/0.177	0.144/0.171	0.213/0.218
No. atoms	1246	1217	1309
Protein	954	925	1064
Ligand/ion	25/12	29/16	36/0
Water	255	247	209
B-factors			
Protein	16.24	15.87	17.12
Ligand/ion	22.35 /28.04	14.97 /42.54	13.67/0
Water	34.76	36.02	29.53
R.m.s deviations			
Bond lengths (Å)	0.004	0.004	0.004
Bond angles (º)	1.044	1.085	1.240

*Highest resolution shell is shown in parenthesis.

Table S5. Data collection and refinement statistics for BRD4-BD1 and BRD2-BD2 X-ray structures

References

1. Kabsch, W. XDS. *Acta Cryst.* **2010**, *D66*, 125–132.
2. Evans, P. R.; Garib, N. M. How good are my data and what is the resolution? *Acta Crystallogr. D: Biol. Crystallogr.* **2013**, *69*, 1204–1214.
3. Vonrhein, C.; Flensburg, C.; Keller, P.; Sharff, A.; Smart, O.; Paciorek, W.; Womack, T.; Bricogne, G. Data processing and analysis with the *autoPROC* toolbox. *Acta Cryst.* **2011**, D67, 293–302.
4. Collaborative Computational Project, Number *4*. Acta Cryst. *D Biol. Crystallogr*. **1994**, *D50*, *760–763*.
5. Murshudov, G.N.; Vagin, A. A.; Dodson, E. J. Refinement of Macromolecular Structures by the Maximum-Likelihood Method. *Acta Cryst.* **1997**, *D53*, 240-255.
6. Emsley, P.; Cowtan, K. Coot: model-building tools for molecular graphics. *Acta Cryst.* **2004**, *D60*, 2126–2132.
7. Bricogne, G.; Blanc, E.; Brandl, M.; Flensburg, C.; Keller, P.; Paciorek, W.; Roversi, P.; Sharff, A.; Smart, O. S.; Vonrhein, C.; Womack, T. O.; (2022). BUSTER . Cambridge, United Kingdom: Global Phasing Ltd.
